# Soil Microbial Communities Associated With Biodegradable Plastic Mulch Films

**DOI:** 10.3389/fmicb.2020.587074

**Published:** 2020-11-13

**Authors:** Sreejata Bandopadhyay, José E. Liquet y González, Kelsey B. Henderson, Marife B. Anunciado, Douglas G. Hayes, Jennifer M. DeBruyn

**Affiliations:** Department of Biosystems Engineering and Soil Science, University of Tennessee, Knoxville, TN, United States

**Keywords:** plastic mulch, biodegradable plastic, soil microbe community, plastisphere, plastic mulch films, plastic biodegradation

## Abstract

Agricultural plastic mulch films provide a favorable soil microclimate for plant growth, improving crop yields. Biodegradable plastic mulch films (BDMs) have emerged as a sustainable alternative to widely used non-biodegradable polyethylene (PE) films. BDMs are tilled into the soil after use and are expected to biodegrade under field conditions. However, little is known about the microbes involved in biodegradation and the relationships between microbes and plastics in soils. In order to capture the consortium of soil microbes associated with (and thus likely degrading) BDMs, agriculturally-weathered plastics from two locations were studied alongside laboratory enrichment experiments to assess differences in the microbial communities associated with BDMs and PE films. Using a combination of amplicon sequencing and quantitative PCR (qPCR), we observed that agriculturally-weathered plastics hosted an enrichment of fungi and an altered bacterial community composition compared to the surrounding soil. Notably, *Methylobacterium*, *Arthrobacter*, and *Sphingomonas* were enriched on BDMs compared to non-biodegradable PE. In laboratory enrichment cultures, microbial consortia were able to degrade the plastics, and the composition of the microbial communities was influenced by the composition of the BDMs. Our initial characterization of the microbial communities associated with biodegradable plastic mulch films, or the biodegradable “plastisphere,” lays the groundwork for understanding biodegradation dynamics of biodegradable plastics in the environment.

## Introduction

Plastic mulch films provide several benefits to fruit and vegetable production, such as reduced weed pressure (Martín-Closas et al., 2017), improved moisture conservation (Kader et al., 2017), and modified soil temperatures (Kasirajan and Ngouajio, 2012). Most plastic films are comprised of polyethylene (PE; Kasirajan and Ngouajio, 2012), which is poorly biodegradable. With limited recycling options, PE waste generally ends up in landfills or is burned (Shogren and Hochmuth, 2004; Ingman et al., 2017), which can lead to soil, groundwater, and atmospheric pollution (He et al., 2014; Steinmetz et al., 2016). Biodegradable plastic mulch films (BDMs) may be a sustainable alternative to PE films. BDMs are made of polymers derived from, or mimicking, those present in bacteria or plants; thus, microbes may have the metabolic capacity to degrade them. Microbes acting on BDMs secrete extracellular depolymerases, which break down the complex polymers into simpler oligomeric and monomeric units (Brodhagen et al., 2015). The latter could then be assimilated by microbes and ultimately converted to biomass or respired (Kyrikou and Briassoulis, 2007). Common biobased polymers used in BDMs include polylactic acid (PLA) and starch; some experimental films have also been manufactured using polyhydroxyalkanoates (PHAs). Fossil-fuel-derived biodegradable polymers used in BDMs include poly(butylene succinate adipate) (PBSA) and copolyesters such as poly(butylene-adipate-co-terephthalate) (PBAT). Commercially-available BDMs are mainly polyesters, sometimes blended with starch. The lower energy of activation for hydrolysis of ester linkages is fundamental to the biodegradability of BDMs compared to polyolefins such as PE (Brodhagen et al., 2015). Consequently, degradation of BDMs can be mediated by microbial hydrolases such as esterases (lipase and cutinase) and proteases. Since natural polyesters such as cutin and suberin structurally resemble plastic polyesters in BDMs, it is not surprising that many of the enzymes reported to degrade these polymers are cutinases (Brodhagen et al., 2015).

Biodegradable plastic mulch films are designed to be tilled into the soil after use, alleviating the disposal issues associated with PE. However, biodegradation of BDMs in the field can be unpredictable. While we accept that microbial biodegradation is the ideal fate of BDMs, there is a considerable knowledge gap regarding the mechanisms of biodegradation, including the specific microbial taxa involved (Bandopadhyay et al., 2018). Among the few studies that have been done, the majority have focused on culturing single strains of bacteria or fungi (Bailes et al., 2013; Moore-Kucera et al., 2014), which may neglect organisms with degradation capabilities that are not easily cultured in the lab. There may also be potential interactions between microorganisms that cannot be inferred from single-strain experiments. In addition, research in this field has focused on degradation of pure polymers under laboratory conditions (Chen et al., 2003; Kunioka et al., 2009; de Eugenio et al., 2010; Shah et al., 2014). However, BDM formulations consist of mixtures of different polymers along with other additives such as fillers, plasticizers, and dyes, and the microbial interactions with these mixtures have not been well documented. Thus, the highly idealized lab studies done to date may not always mimic BDM biodegradation dynamics in the field.

While several studies have characterized microbial communities in soils near BDMs (Koitabashi et al., 2012; Li et al., 2014b; Muroi et al., 2016), none have characterized the communities directly associated with the plastic films. Additionally, it is unknown if the microbes colonizing BDMs would be similar to those that colonize PE film. Moreover, the dependence of random vs. non-random surface colonization on plastic composition is not understood. It is not clear, for example, if different chemical compositions of plastic mulches might select for different taxa in the colonizing communities. The few studies that have compared BDMs and PE in terms of soil microbes have reported increased microbial abundances, respiration rates, and potential extracellular enzyme activity rates in soil under BDMs compared to PE mulches (Moreno and Moreno, 2008; Li et al., 2014a; Barragán et al., 2016; Ma et al., 2016), implying that BDMs enhance microbial activity in soils. Further examination of microbial communities directly associated with field-deployed plastic mulch films will help further our knowledge of the types of microbes that potentially degrade BDMs *in situ*.

In this study, our goal was to identify the BDM “plastisphere,” i.e., microbial communities associated with plastic mulch films. The objectives of this study were to characterize the microbial communities associated with BDMs both in controlled laboratory enrichment cultures as well as on agriculturally-weathered plastic mulches and soil from the field, so that we can understand the taxa enriched on field-deployed BDMs. We hypothesized that *Proteobacteria*, *Actinobacteria*, *Firmicutes*, and *Ascomycota* would be enriched on BDMs compared to PE; all of these phyla include candidates with demonstrated polymer degradation capabilities. We further hypothesized that the microbial communities enriched on BDMs would differ between films of different composition. Our work demonstrates bacterial taxa in close association to BDMs and highlights potential BDM degraders *in situ* and *in vitro*, co-occurrence of bacterial and fungal taxa *in situ* that might be involved in BDM degradation, and specific taxa enriched on BDMs. With our results, we hope to lay a foundation for future work on BDM degradation mechanisms in soil.

## Materials and Methods

### Plastic Mulch Films

Four BDMs were used in this study. Three of these were commercially available: BioAgri®, Naturecycle, and Organix™; the fourth was an experimental PLA/PHA film. The three commercial films are PBAT-based, and BioAgri, and Organix are composed of MaterBi® and ecovio® feedstocks, respectively. In addition to the four BDMs, the field study included a fully biodegradable paper (cellulosic) mulch (WeedGuardPlus®, positive control) and a non-biodegradable PE film (negative control). The major constituents, properties, and manufacturers of these mulch films are in Supplementary Table S1. Unweathered BDMs were stored in the dark at room temperature to be used as controls for thermogravimetric analysis (TGA; described below).

### Enrichment Cultures

#### Enrichment Culture Experimental Design

Soil used for the enrichment study were collected in May 2016 from the East Tennessee Research and Education Center (ETREC) located in Knoxville, TN (35° 54' 15.7176'' N, 83° 57' 13.7484'' W). Soils were collected from test plots where biodegradable and PE mulches were being tested under pie pumpkin (*Cucurbita pepo* L.) as a test crop, with full experimental details described in related manuscripts (Ghimire et al., 2018; Sintim et al., 2019). At the time of collection, soils were exposed to mulches for one complete field season (May 2015–2016). The soil at Knoxville is a sandy loam (59.9% sand, 23.5% silt, and 16.6% clay), classified as a fine kaolinitic thermic Typic Paleudult. Soil used in the study was collected from one of the four replicate plots covered with the biodegradable mulch BioAgri, which was tilled into the soil on 14 October 2015. About 30 subsamples of soil collected at 10 cm depth were composited and stored at −20°C until enrichment cultures were performed.

Enrichment cultures were set up in June 2016 in Wheaton™ media bottles with butyl septa to enable headspace carbon dioxide (CO_2_) sampling for respiration measurements. Soil (30 g) was vortexed with 270 ml 1X phosphate buffered saline (PBS) resulting in a 10:1 v/v dilution. The 1X PBS solution contained 137 mM NaCl, 2.7 mM KCl, 10 mM Na_2_HPO_4_, and 1.8 mM KH_2_PO_4_ adjusted to a final pH of 7.4. The resultant soil extract was used for inoculation of the enrichment cultures. Plastic samples were obtained from the rolls received from the manufacturers (i.e., unweathered), cut into 4 × 4 cm^2^ pieces and sterilized by UV irradiation in a biosafety cabinet for 1 h on each side following a published protocol with modifications (Bailes et al., 2013). Sterilized films were kept in sterile containers inside the hood until they were added to culture bottles. Three pieces of each BDM were put into each bottle. For each BDM used, bottles were either inoculated with 5 ml of the soil extract or left uninoculated by adding only 5 ml of 1X PBS. Soil extract (5 ml) was added to M9 minimal media (45 ml) in inoculated bottles, resulting in a final dilution of 10^−2^ v/v. Uninoculated bottles received BDMs and media but no soil extract. A negative control containing only media (no soil inoculum or BDMs) was also used. All treatments and controls were done in triplicate. Two separate enrichment incubations were set up: one set was incubated for 3 weeks and the other set for 6 weeks. The bottles were incubated in an incubator shaker (New Brunswick Scientific Co. Inc., Edison, NJ, United States) at 30°C with shaking at 120 rpm in the dark. BDM pieces were collected after 3 and 6 weeks of incubation: one BDM piece was used to conduct confocal microscopy to visualize live/dead microbes adhering to BDMs; a second piece was used to extract DNA for sequencing of adherent soil microbes; and the third piece was used to assess material properties of the plastic. The plastics used for imaging and sequencing were not cleaned; the plastic used for testing material properties was cleaned gently.

#### BDM Biodegradation Assessment

Carbon dioxide evolution served as a metric of biodegradation of BDMs. A syringe was used to collect 0.5 ml aliquots of air headspace in the culture bottles through butyl septa cap. CO_2_ concentrations were determined using an infrared gas analyzer (LI-820, Licor Inc., Lincoln, NE, United States). Gas standards (1,000, 10,000, and 50,000 ppm) were used to create standard curves. Sample CO_2_ ppm values were then derived from standard curves and final values reported as cumulative CO_2_ (ppm) production over time.

#### Confocal Microscopy

Plastics sampled after 3- and 6-week incubations were stained using a LIVE/DEAD® BacLight™ Bacterial Viability Kit (Invitrogen™ Molecular Probes™, Eugene, OR, United States). The manufacturer’s protocol was modified and adjusted for optimal staining of plastic pieces: 10 μl of Component A (SYTO 9 dye, 1.67 mM/Propidium iodide, 1.67 mM, 300 μl solution in DMSO) was mixed with 10 μl of Component B (SYTO 9 dye, 1.67 mM/propidium iodide, 18.3 mM, 300 μl solution in DMSO). Components were mixed thoroughly in a microcentrifuge tube. 3 μl of the dye mixture was mixed with 1 ml of M9 minimal media. The reagent mixture contributed roughly 0.3% DMSO to the staining solution. The plastic piece to be visualized was mounted on a glass slide and the mixture was poured onto it and covered with an aluminium foil. Slides were incubated at room temperature in the dark for 15 min. After incubation, the plastic was rinsed off gently with M9 minimal media to avoid background plastic being stained. A drop of mounting oil was placed on the plastic piece and covered with a cover slip. Imaging was completed using a Leica™ Scanning Laser Confocal System at the University of Tennessee, Advanced Microscopy and Imaging Center. About 5–10 fields of vision were scanned. Scale bars were added to images using ImageJ® software (Schneider et al., 2012).

#### Esterase Assay

An absorbance-based esterase assay was conducted using a modified version of the lipase-esterase assay described by Margesin et al. (2002). The protocol, which was initially developed for test tube scale soil assays, was modified and standardized for a 1.5 ml- deep 96-well plate assay. Saturation curves were conducted to determine optimal volume of the para-nitrophenyl butyrate substrate needed to add to the soil-buffer system: 200 μl and 250 μl substrate was deemed optimal for the 3- and 6-week samples. Around 100 μl of spent media extract from each enrichment bottle was added to triplicate wells in a 96-well plate. Standard curves [0–100 μg ml^−1^ para-nitro phenol, (pNP)] were separately run using each of the spent media extracts in the same plate to account for any background signal. The same protocol was followed for the standard and test samples. Phosphate buffer (adjusted to total volume of 500 μl reaction mixture) was added to the spent media and the plate was pre-warmed in water bath at 30°C for 10 min. The previously determined optimal volume of para-nitrophenyl butyrate substrate was then added to each well. For standard curves, the appropriate volume of pNP was added. After addition of substrates and standards, well contents were mixed and plates incubated at 30°C for exactly 10 min. The reaction was stopped by cooling on ice for 10 min. Finally, the plate was centrifuged at 2000 rcf at 4°C for 5 min. Supernatants were pipetted into a new 96-well clear plate and absorbance of the released pNP in samples was read at 400 nm on a microplate reader (Biotek® Synergy H1).

#### Isolation of BDM Degrading Bacteria

Isolation of BDM-degrading bacteria was done using the spent enrichment culture media from the 6-week incubated bottles following the method of Bailes et al. (2013) with modifications. Inoculum was taken from the spent media in culture bottles and spread on Difco™ Noble Agar (BD, Sparks, MD, United States) plates with sterilized BDM films as the sole carbon source. Colonies were selected from the edge of the films and then re-streaked several times onto M9 minimal media agar plates with plastic as the sole carbon source. Controls included M9 minimal agar plates with and without glucose but no added plastic. DNA was extracted from these isolates using MoBio™ UltraClean Microbial DNA isolation kit (now Qiagen™, Hilden, Germany) per manufacturer’s instructions. 16S rRNA genes were PCR-amplified and sequenced using Sanger sequencing at the University of Tennessee Genomics Lab.

#### Thermogravimetric Analysis

BioAgri and Organix plastics were sampled from enrichment culture bottles after 6 weeks and gently rinsed with sterile water to remove adherent microbial biofilm and dried at room temperature for TGA. Approximately 2 mg of these BDMs (~5 mm square) were cut and placed onto platinum TGA pans. Samples were then heated at a constant rate of 10°C per minute from room temperature (25°C) to 600°C using TGA 550 thermogravimetric analyzer (TA Instruments, New Castle, DE, United States). Onset temperatures of degradation processes, weight remaining (%), and rate of weight loss were determined. Results were compared to indoor-stored off-the-roll BDMs.

#### Attenuated Total Reflectance-Fourier Transform Infrared Spectroscopy

The attenuated total reflectance-fourier transform infrared spectroscopy (ATR-FTIR) spectra were obtained using IRAffinity-1 spectrometer (Shimadzu Co., Tokyo, Japan) equipped with a single reflection ATR System (MIRacle ATR, PIKE Technologies, Madison, WI, United States) with a resolution of 2 cm^−1^. Sixteen scans per spectrum were recorded with a wavelength range between 4,000 and 600 cm^−1^ for each plastic sample.

#### Gel Permeation Chromatography

Plastic samples (20 mg) were dissolved in 5 ml chloroform (CHCl_3_) and magnetically stirred for 1 h and centrifuged at 10,000 rpm (6,149 × *g*) for 1 min. Solution was passed through a 1.0 and 0.2 μm nylon filters to remove CHCl_3_-insoluble materials. Two hundred microliters of filtered solution was injected into a gel permeation chromatography (GPC) system equipped with HPLC system (Shimadzu Columbia, MD, United States), equipped with a model Mark IIII evaporative light scattering detector (ELSD; WR Grace, Deerfield, IL United States) and a 300 × 7.5 mm ID PL Gel mixed D column purchased from Agilent (Santa Clara, CA United States), using a flow rate of 0.8 ml/min and a runtime of 13 min.

### Field Study

#### Field Sites

Field samples were collected from two experimental stations with ongoing BDM plot experiments: ETREC in Knoxville, TN, and the Northwestern Washington Research and Extension center (NWREC) at Mount Vernon, WA (48° 26' 20.8968'' N, 122° 23' 14.8524'' W). These two sites had different climates and soil types. The soil at Knoxville is a sandy loam (59.9% sand, 23.5% silt, and 16.6% clay) classified as a fine-loamy, mixed, subactive, thermic Typic Hapludult, or Haplic Alisol. The soil at Mount Vernon is a silt loam (14.2% sand, 69.8% silt, and 16% clay), classified as a fine-silty mesic Fluvaquentic Endoaquept or Haplic Fluvisol (Sintim et al., 2019). The mean soil pH in Knoxville and Mount Vernon was 6.03 ± 0.36 and 6.24 ± 0.11, respectively. Both field sites were set up as randomized complete block design experiments with four replications of seven main plot treatments totaling to 28 plots at each location. These treatments included four BDM treatments, a PE mulch, a cellulose mulch, and a no-mulch (bare ground) treatment. Full experimental design and details can be found in Ghimire et al. (2018) and soil physiochemical properties are reported in Sintim et al. (2019). Summary of weather data for 2016 is reported in Supplementary Table S2 with data over 2 years reported in Sintim et al. (2019). The present study reports data on soil and plastic mulch samples collected in Fall of 2016 after a complete growing season using pie pumpkin, *Cucurbita pepo* cv. Cinnamon girl, as a test crop in both TN and WA.

#### Soil Sampling

Soil samples were collected from all 28 plots at ETREC and NWREC in September 2016, after harvest but before mulches were removed or tilled into soil, as described in Sintim et al. (2019). Briefly, 30 subsamples of 10-cm deep soil were obtained from the middle of five rows of each plot using sterilized soil augers and composited. Soils were transferred to the laboratory in Ziplock® bags, then 20 g of each soil sample was aliquoted into sterile Whirlpak® bags and stored at −80°C for DNA extraction.

#### Agriculturally-Weathered Mulch Sampling

Agriculturally-weathered mulches were collected from both ETREC and NWREC field sites at the end of the growing season in 2016 (October) from all mulched plots (six mulch treatments with four replicate plots for each, for a total of 24 plots). Six pieces of mulches approximately 8 cm^2^ were cut from the middle row of each plot using scissors sterilized with 70% ethanol to prevent cross contamination. Any clumps of adherent soil were removed. The six pieces were placed in a sterile Whirlpak® bag and immediately placed on ice. Mulch samples were stored at −20°C for DNA extractions.

### Microbial Community Analysis for Lab and Field Study

#### DNA Extraction, Quantification, and qPCR

DNA extraction from soil and plastic samples was completed using the Qiagen™ (formerly MoBio™) PowerLyzer Power Soil DNA isolation kit with inhibitor removal technology. DNA extraction from soil samples were completed per the manufacturer’s instructions. However, for the mulch samples the protocol was optimized differently. Two pieces of mulch (0.7 × 0.7 cm^2^) were cut from each of the six pieces of agriculturally-weathered mulches sampled from the field resulting in 12 pieces total. Four pieces went into one bead beating tube provided in the PowerLyzer Power Soil DNA kit (Qiagen™), and DNA extractions performed according to manufacuter’s protocol. Triplicate DNA extractions (i.e., four pieces of plastic per tube) were performed for each plot and pooled prior to DNA quantification, quantitative PCR (qPCR), and sequencing. BDMs retrieved from enrichment culture bottles were similarly cut into small square pieces (0.7 × 0.7 cm^2^ each) using sterile scissors and forceps inside a biosafety cabinet. DNA extraction was done as described for the agriculturally-weathered plastics.

DNA concentration was assessed using a Quant-It PicoGreen dsDNA Quantification Kit (Invitrogen™) per manufacturer’s instructions. Mean DNA concentration from soil samples were 12 ng μl^−1^ DNA; mean DNA concentration from plastic samples was 3.3 ng μl^−1^. Assay standard curves all had *R*^2^ > 0.99.

16S rRNA (bacteria) and internal transcribed spacer (ITS; fungi) gene copy numbers were quantified from plastic and soil DNA samples *via* qPCR using Femto™ Bacterial/Fungal DNA quantification kit. Known amounts (ng) of bacterial and fungal DNA were provided in the kit as standards. These were converted to copy numbers and used to generate a standard curve to calculate copy numbers in our samples. Mean qPCR efficiencies were approximately 85 and > 90% for the bacterial and fungal assays, respectively. Assay standard curves all had *R*^2^ > 0.98. Bacterial to fungal abundance ratios were calculated to look for relative enrichment of bacteria vs. fungi in soil and plastic samples.

#### 16S rRNA Amplification and Sequencing

16S rRNA amplicon sequencing of extracted DNA samples was conducted by the Genomic Services Laboratory (GSL) at Hudson Alpha, Huntsville, AL. DNA samples were shipped in 96-well plates with dry ice, and GSL performed all library preparation and sequencing. V4 region was amplified using primers 515F (GTGCCAAGCAGCCGCGGTAA) and 806R (GGACTACHVGGGTWTCTAAT; Caporaso et al., 2011). The first PCR was run with V4 amplicon primers, Kapa HiFi master mix, and 20 cycles of PCR. All aliquots and dilutions of the samples were completed using the Biomek liquid handler. PCR products were purified and stored at −20°C until further processing was completed. The PCR indexing was later completed for the 16S (V4) amplicon batch. Products were indexed using GSL3.7/PE1 primers, Kapa HiFi master mix, and 12 cycles of PCR. Products were purified using magnetic beads using the Biomek liquid handler. Final libraries were quantified using PicoGreen. A subset of libraries was profiled on the Agilent DNA1000. The amplified 16SrRNA genes were sequenced using 250PE (paired-end) reads on an Illumina MiSeq platform. Data was transferred from the GSL to University of Tennessee *via* BaseSpace. Sequence data was processed using Mothur v.1.39.5 (Schloss et al., 2009) following the standard operating protocol for MiSeq libraries. First, primers were trimmed from the sequences. This was followed by making contigs of the paired end reads, and removing sequences with ambiguous bases and long homopolymers. SILVA v102 was used as a reference alignment by customizing it to our region of interest which was the 16S rRNA gene. First, an *Escherichia coli* 16S rRNA gene sequence was obtained and trimmed to start and end with our primer pair. The resulting fasta file was aligned to the SILVA reference file. The summary of the alignment provided the start and end positions (or the coordinates) to use in pcr.seqs command to trim the SILVA alignment file to our region of interest, i.e., the V4 region. After alignment to SILVA reference database, sequences were filtered to remove overhangs at both ends, and sequences de-noised by pre-clustering them up to two differences between sequences. Chimeras were removed using VSEARCH algorithm. Unwanted sequences classified as *Eukaryota* and *Arachaeota* were removed. Sequences were binned into phylotypes according to their taxonomic classification and cut off set at the genus level. A consensus taxonomy for each operational taxonomic unit (OTU) was generated using the RDP reference database version 9. The resulting OTU table and taxonomy file were imported into R for further analyses.

#### 18S rRNA Amplification and Sequencing

18S rRNA sequencing of the agriculturally-weathered plastic samples was completed by amplifying the V4 region using primers 574F (CGGTAATTCCAGCTCYV) and 1132R (CCGTCAATTHCTTYAART). The 18S amplicon PCR products were used at an input of 10 μl of gDNA. Kapa HiFi master mix and 20 cycles of PCR were used. PCR products were purified using magnetic beads on the Biomek. The indexing PCR reaction was then set up using GSL3.7_I7 primers and the truncated PE1 primer. PCR products were then again purified using magnetic beads on the Biomek. 18S libraries were quantified using PicoGreen as described above. A subset of final libraries was analyzed on an Agilent DNA1000 chip. Kapa qPCR was completed for samples and a MiSeq 300 bp PE run was completed. The fastq files from the run were converted to a “.files” format, which was then used to trim off primers, make contigs, remove sequences with ambiguous bases and long homopolymers using Mothur v.1.39.5 (Schloss et al., 2009) as described for the 16S libraries. SILVA v132 was used as reference alignment by customizing it to 18S rRNA following the same workflow as for the 16S rRNA gene. Instead of using *E. coli*, *Saccharomyces cerevisiae* sequence was used to trim to begin and end with the primer pair used for 18S sequencing. The *S. cerevisiae* sequence was saved as a fasta file and aligned to the SILVA reference file to get the start and end coordinates to use to trim the SILVA reference to the V2 amplicon region.

### Statistical Analysis

For the lab study, three-way ANOVAs were conducted to assess differences in 16S rRNA, ITS gene abundances as well as 16S/ITS ratios between times of incubation (3- and 6-week incubations), inoculated and uninoculated samples, and between plastic treatments. In case of significant interaction effects, one-way ANOVAs were done to report simple main effects. Tukey *post hoc* tests were conducted following one-way ANOVAs with significant differences. For the field study, a mixed model ANOVA was used to analyze differences in 16S/ITS ratios between locations, soil and plastic communities, and plastic treatments; block was used as a random effect with all other parameters being fixed effects. Simple main effects were also evaluated for the field 16S/ITS ratios for all the parameters (location, soil/plastic, and treatment) using one-way ANOVAs. T-tests were conducted to assess differences in CO_2_ evolved between inoculated and uninoculated lab enrichments, as well as for 16S/ITS ratios between agriculturally-weathered BDMs and bulk soil for Fall 2016. All statistics were done in R v. 3.4.0 (R Core Team, 2018).

Bray-Curtis distances of microbial community composition were calculated using the vegan package (v 2.4-3) in R platform. The distances were then visualized using non-metric multidimensional scaling (NMDS) plots using phyloseq package in R. To determine whether significant differences existed between treatments in bacterial and eukaryotic community compositions, a permutational multivariate analysis of variance (PERMANOVA) was performed using the ADONIS function implemented in the R statistical platform (version 3.4.0), based on the Bray-Curtis dissimilarity matrix. Similarity percentage analyses (SIMPER) was computed in R, revealing the most influential OTUs driving differences in bacterial and eukaryotic community composition between soil and agriculturally-weathered plastic, locations, and between mulch treatments. Difference in relative abundances of bacterial and eukaryotic taxa between treatments was determined using Kruskal-Wallis rank sum non-parametric test (kruskal.test in R).

For alpha diversity values for BDMs exposed to lab enrichment, agriculturally-weathered mulch and bulk soil, number of observed OTUs (richness) and inverse Simpson indices (diversity) of bacterial and eukaryotic communities were calculated in R. The libraries were subsampled with replacement (subsampling was done to the minimum number of reads) and repeated 100 times to estimate the species abundance, while standardizing sampling effort; the estimates were then averaged from each trial. Three-way ANOVAs were conducted to test for interaction effects for the lab study. In case of significant interaction effects, a one-way ANOVA was used in R to test for simple main effects; simple main effects for lab study were tested using time of incubation, inoculated/uninoculated treatments, and mulch treatments as factors. Tukey *post hoc* tests were conducted following one-way ANOVAs on treatments. For the field study, a mixed model ANOVA was done to account for fixed effects and random effects similar to the analysis for 16S/ITS ratios. Simple main effects were also evaluated using one-way ANOVAs for the alpha diversity estimates obtained from the field study using location, soil/plastic treatments, and mulch treatments as factors, followed by a Tukey *post hoc* test. All sequence reads were scaled to minimum library size before analysis was performed. Rarefaction curves were obtained using Chao1 (Chao, 1984) estimates and observed number of OTUs in R. The richness estimator, Chao1, is based on distribution of singletons and doubletons and attempts to account for unobserved OTUs by extrapolating from the observed abundances.

### Data Availability Statement

Sequence data can be accessed at Sequence Read Archive under BioProject ID PRJNA631499 for the plastic samples. The sequences for the soil samples can be found under BioProject ID PRJNA564156 (Bandopadhyay et al., 2020).

## Results

### Biodegradation of BDMs in Enrichment Cultures

**Figure 1:**
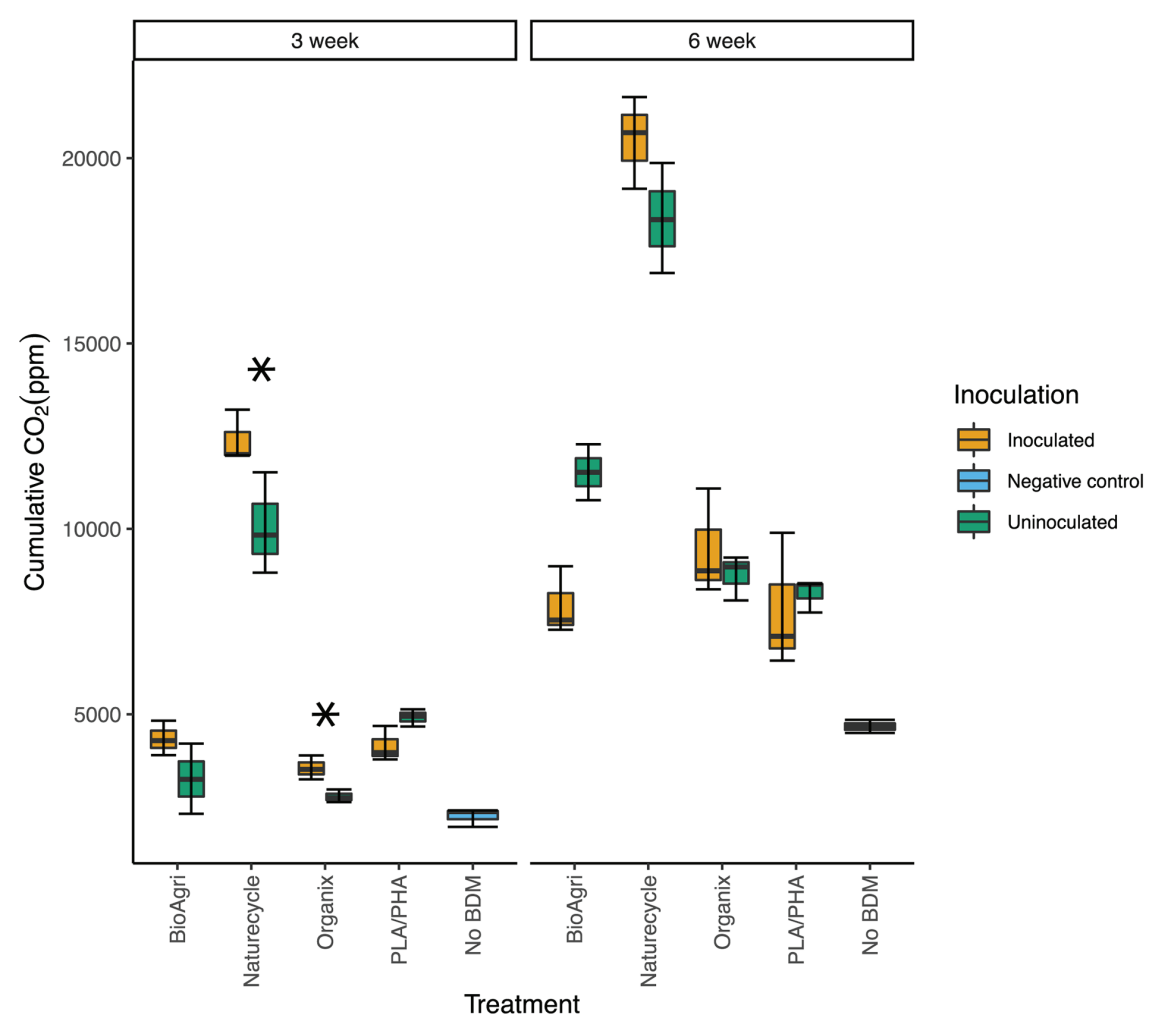
Cumulative carbon dioxide (CO_2_) measured in enrichment culture headspace gas after 3- and 6-week incubations. The lower and upper hinges of the boxplots correspond to the 25th and 75th percentiles and the middle of the box denotes the median. Whiskers denote 1.5 times the inter-quartile range. Asterisks indicate significant differences between uninoculated and inoculated enrichments from a *t*-test; ^*^p ≤ 0.05.

Increased CO_2_ production in enrichment cultures indicated biodegradation of BDMs. All inoculated BDM treatments showed significantly higher CO_2_ evolution than the negative control (no BDM) for both 3- and 6-week incubated cultures (*p* ≤ 0.05). Inoculated bottles had significantly higher CO_2_ (*p* ≤ 0.05) than uninoculated samples for Naturecycle and Organix treatments after 3-week incubations (Figure 1). After 6 weeks, cumulative CO_2_ evolution for all treatments was greater than the 3-week incubation (Figure 1); however, there were no significant differences between inoculated and uninoculated samples.

Biodegradation of BDMs was also indicated by changes in molecular properties of plastic. Thermogravimetric analyses showed changes in thermal properties of BioAgri (PBAT and starch) and Organix (PBAT and PLA) after 6 weeks (Supplementary Figure S1). The heating stage attributable to starch (Supplementary Figure S1B) shows slight evidence of depolymerization; both inoculated and uninoculated BDMs had slightly shifted its peak heating stages to lower temperatures. Inoculated Organix had a more pronounced shift of the PBAT heating stage peak (centered at 400°C) compared to the uninoculated Organix treatment (Supplementary Figure S1D). TGA was not performed for Naturecycle as per the request of the manufacturer. GPC data (Supplementary Table S3) suggests a decrease of molecular weight for PBAT in the inoculated BioAgri treatment. Fourier Transform Infrared Spectroscopy (FTIR) analysis of BioAgri suggests a decrease of the OH stretching region (3,600–3,200 cm^−1^) and C=O stretching (1,648 cm^−1^) for both inoculated and uninoculated treatments relative to the initial, both bands of which are attributable to starch (Hayes et al., 2017; Supplementary Figure S2A). For Naturecycle, as with BioAgri, there is some evidence that starch may be the preferred carbon source (e.g., decrease of OH stretching region, 3,800–3,200 cm^−1^ for inoculated and uninoculated treatments; Supplementary Figure S2B) but the change in spectra from the initial was much less pronounced than observed for BioAgri. There was no change in the region 1,800–1,200 cm^−1^ attributable to the polyester (Hayes et al., 2017; Supplementary Figure S2B). FTIR and GPC analyses did not reveal any changes for PLA/PHA indicative of biodegradation. Organix, unlike BioAgri and Naturecycle, contains starch at a much lower concentration. However, the polyesters in its feedstock, ecovio®, are similar with Naturecycle and BioAgri with PBAT being the major shared component. For Organix, the most major changes in the FTIR spectra were decreases in the -CH2- bend, C-O stretching and OH bending regions (1,500–800 cm^−1^). The greatest change, similar to BioAgri, occurred for the uninoculated treatment, while the spectrum of the inoculated treatment changed minimally from the initial.

Evidence of microbial colonization of films in inoculated and uninoculated enrichments was seen in confocal images using a LIVE/DEAD® Bacterial Viability kit (Supplementary Figure S3). Live cells stained green, while dead cells and fungal hyphae stained red. As expected, inoculated BDMs showed qualitatively denser microbial colonization than uninoculated BDMs. Propidium iodide used as part of the staining kit also stains fungal hyphae, which could be seen on the plastics (e.g., Naturecycle-inoculated treatment in Supplementary Figure S3).

The potential activity of extracellular esterase was assayed in the inoculated media following the 3- and 6-week incubation. Esterase activity significantly increased over time for PLA/PHA and BioAgri (Supplementary Table S4). After 3 weeks of incubation, the greatest esterase activities were observed for PLA/PHA and Organix with 2.14 ± 0.06 and 1.8 ± 0.46 μg pNP released ml^−1^ min^−1^ (Supplementary Table S4), respectively; whereas after 6 weeks, the greatest activity was observed for BioAgri and PLA/PHA with activities of 3.05 ± 0.31 and 2.73 ± 0.13 μg pNP released ml^−1^ min^−1^ closely followed by Naturecycle with an activity of 2.63 ± 0.21 μg pNP released ml^−1^ min^−1^.

### Bacterial Communities Enriched on BDMs in Laboratory Cultures

#### Effect of Incubation Length (3 vs. 6 weeks)

When all treatments were analyzed together, there was no significant difference in bacterial or fungal abundances between the 3- and 6-week incubations (Supplementary Table S5). However, there were slight differences between 3- and 6-week incubations for individual treatments demonstrated *via* one-way ANOVAs (Supplementary Table S6). For example, inoculated Organix had significantly greater bacterial abundance at 3 weeks (Supplementary Figure S4; Supplementary Table S6; *p* ≤ 0.05), but uninoculated Organix had significantly greater ITS gene abundance at 6 weeks (Supplementary Figure S4; Supplementary Table S6; *p* < 0.001). Inoculated PLA/PHA treatment showed significantly greater 16S/ITS gene abundance ratio at 6-week compared to 3-week incubation (*p* ≤ 0.05; Supplementary Table S6). Microbial community composition and diversity was unaffected by incubation length. Rarefaction curves of the sequencing libraries are shown in Supplementary Figure S5. Neither richness nor diversity was significantly different between the 3- and 6-week incubations (Supplementary Tables S7–S9). Similarly, no significant differences were seen in bacterial community composition between 3- and 6-week incubation times for each treatment (in inoculated and uninoculated enrichments; Supplementary Table S10).

#### Comparison Between Inoculated and Uninoculated Enrichment Cultures

Microbial gene copy abundances were significantly different between the soil-extract inoculated and uninoculated samples. Naturecycle had significantly greater 16S rRNA (*p* ≤ 0.01; Supplementary Figure S4A; Supplementary Table S11) and ITS (*p* ≤ 0.05; Supplementary Table S11; Supplementary Figure S4B) gene abundances in inoculated treatments after both 3- and 6-week incubations, and a relatively higher proportion of fungi (i.e., lower 16S/ITS ratio). PLA/PHA had significantly greater bacterial abundance (*p* ≤ 0.05) and a greater proportion of bacteria (*p* ≤ 0.01; Supplementary Table S11) on inoculated treatments after 6 weeks. Organix had significantly greater ITS gene abundances on inoculated treatments compared to uninoculated ones (*p* ≤ 0.05) after 3 weeks. No significant change was observed between inoculated and uninoculated treatments for BioAgri.

**Figure 2:**
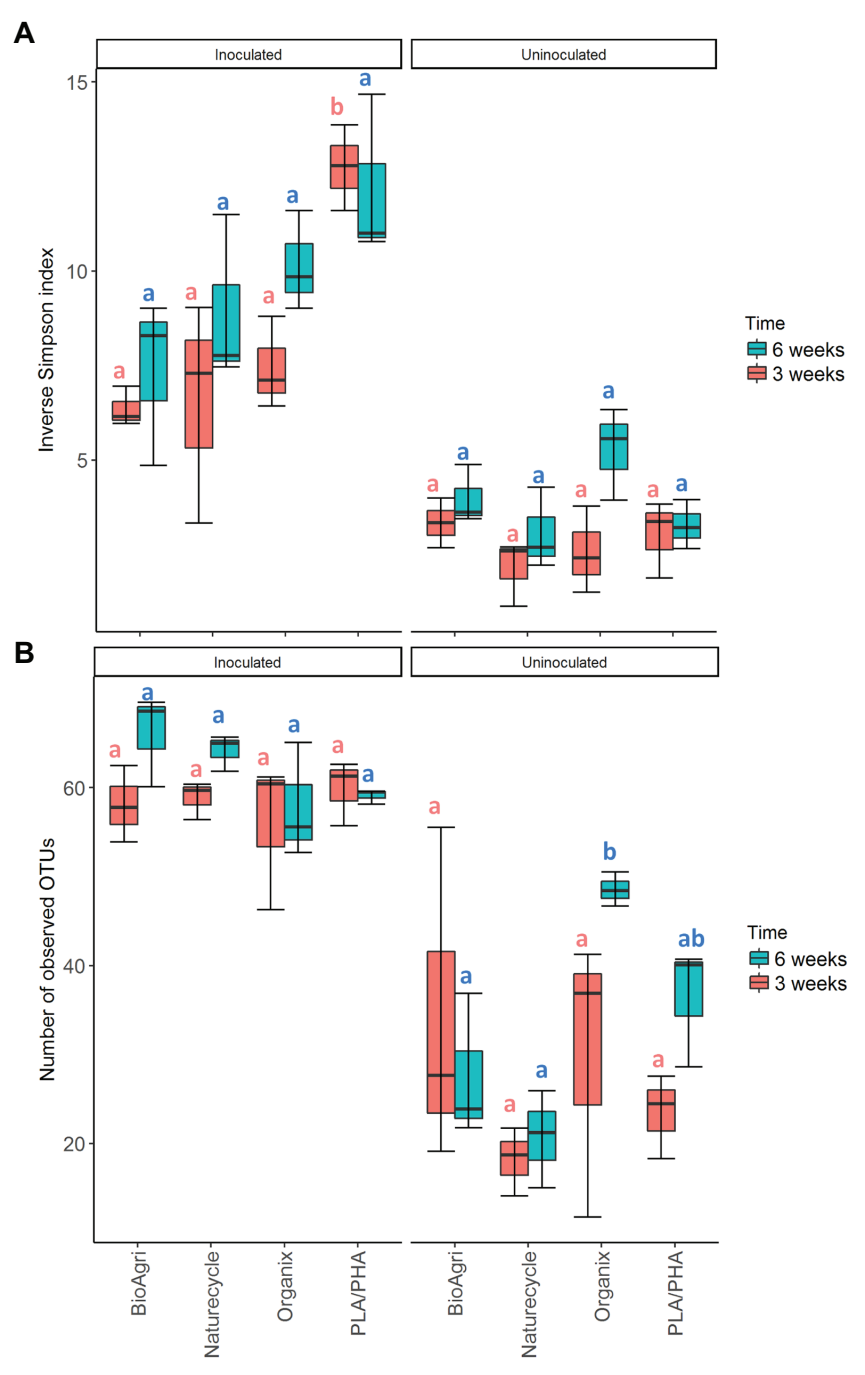
**(A)** Diversity (inverse Simpson index) and **(B)** richness [number of observed operational taxonomic unit (OTU)] for bacterial communities in inoculated and uninoculated enrichments. The lower and upper hinges of the boxplots correspond to the 25th and 75th percentiles and the middle of the box denotes the median at 50th percentile. Whiskers denote 1.5 times the inter-quartile range. Letters indicate Tukey *post hoc* results following a one-way ANOVA. All significances tested at *α* = 0.05.

**Figure 3:**
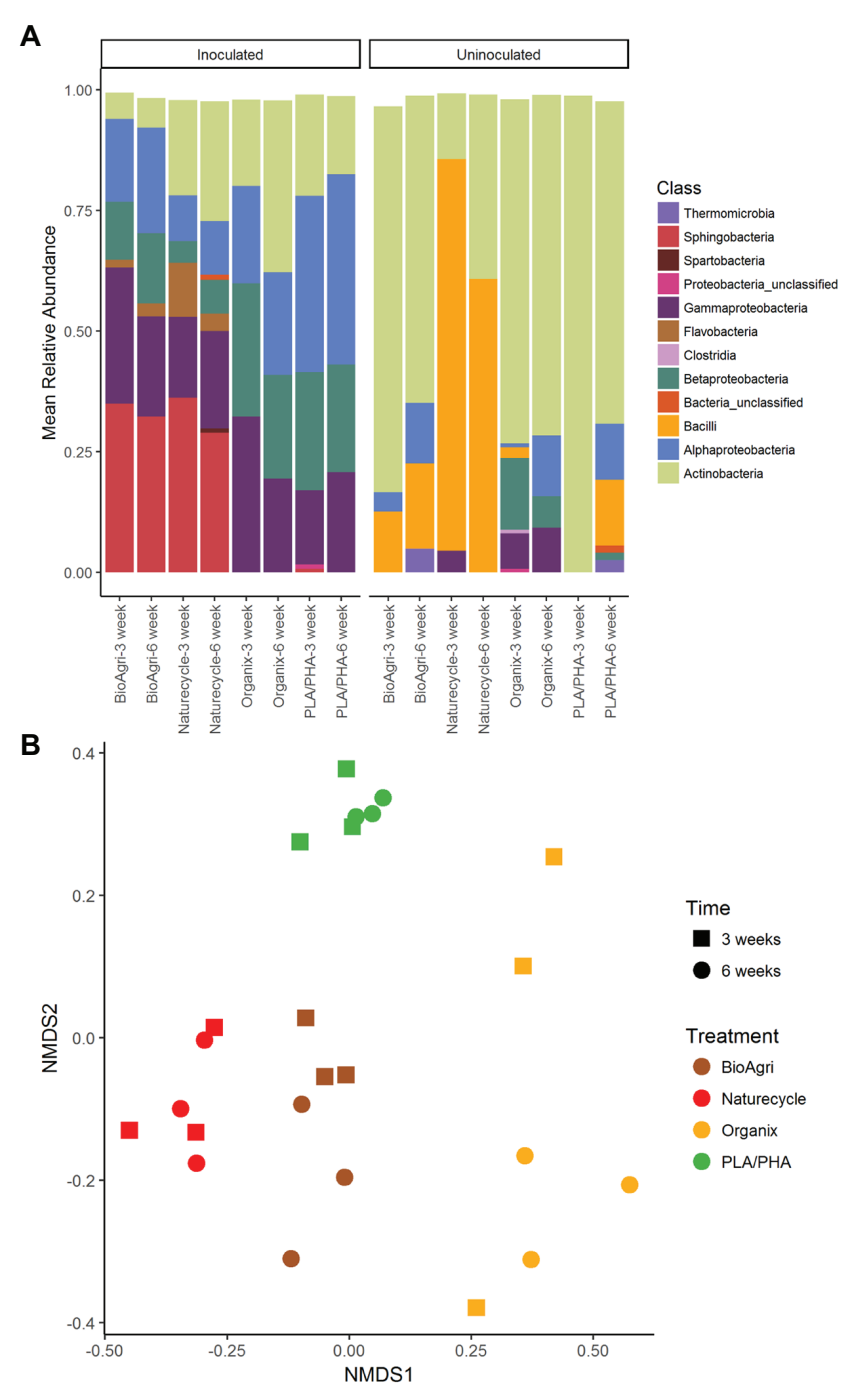
**(A)** Bacterial taxa distribution (class level) on biodegradable plastic mulch films (BDMs) in laboratory enrichment cultures. Mean relative abundances above a cut-off level of 2% are indicated. “Bacteria unclassified” denote taxa with relative abundances above the cut-off level of 2%, but that could not be classified. **(B)** Non-metric multidimensional scaling (NMDS) ordination of bacterial communities on inoculated BDMs. Time refers to incubation time; treatments are four different biodegradable mulch films. NMDS stress value: 0.24.

In addition to the increased microbial abundances, inoculated treatments also had significantly higher richness and diversity estimates compared to uninoculated samples for both 3- and 6-week incubations (Figure 2). The community structure was also significantly different between inoculated and uninoculated samples for both 3-week (*p* = 0.001) and 6-week (*p* = 0.001) incubations (Supplementary Table S10). *Actinobacteria* and *Bacilli* were higher in relative abundance in all uninoculated treatments (Figure 3). *Sphingobacteria*, *Gammaproteobacteria*, *Alphaproteobacteria*, and *Betaproteobacteria* dominated BDM-associated communities in inoculated treatments (Figure 3). To supplement sequencing results, isolates were obtained from media in inoculated culture bottles using agar plates with sterilized BDMs as the sole carbon source (Supplementary Figure S6). Isolates were identified based on 16S rRNA gene sequences as *Streptomyces* sp., *Arthrobacter* sp., *Pseudomonas* sp., *Variovorax* sp., and *Microbacterium* sp. (Supplementary Table S12).

#### Comparison Between Mulch Treatments

There were significant differences in 16S rRNA gene copy abundances between BDM treatments for both 3- and 6-week inoculated incubations (*p* ≤ 0.05; Supplementary Table S13). There were significantly greater abundances of bacteria on Naturecycle and PLA/PHA compared to BioAgri and Organix (Supplementary Table S14; Supplementary Figure S4). There were no significant differences in richness of the bacterial communities; however, we did observe a significantly greater diversity on PLA/PHA after 3 weeks (Supplementary Table S9; Figure 2). For uninoculated treatments, there was an increase in richness on Organix after 6 weeks, but no significant differences in diversity. We observed significant differences in microbial community structure between the different inoculated BDMs (Figure 3B; Supplementary Table S10), with *Gammaproteobacteria*, *Alphaproteobacteria*, *Sphingobacteria*, *Flavobacteria*, and *Betaproteobacteria* enriched on starch-containing BDMs (Naturecycle and BioAgri) and *Gammaproteobacteria*, *Alphaproteobacteria*, and *Betaproteobacteria* enriched on PLA-containing films (Organix and PLA/PHA; Figure 3A). At the genus level, *Sphingobacteria* and *Flavobacteria* were enriched on starch-containing Naturecycle and BioAgri plastics, whereas *Pseudomonas* and *Hydrogenophaga* were enriched on PLA-containing Organix and PLA/PHA plastics (Supplementary Figure S7).

### Bacterial and Eukaryotic Communities Associated With Agriculturally-Weathered BDMs

**Figure 4:**
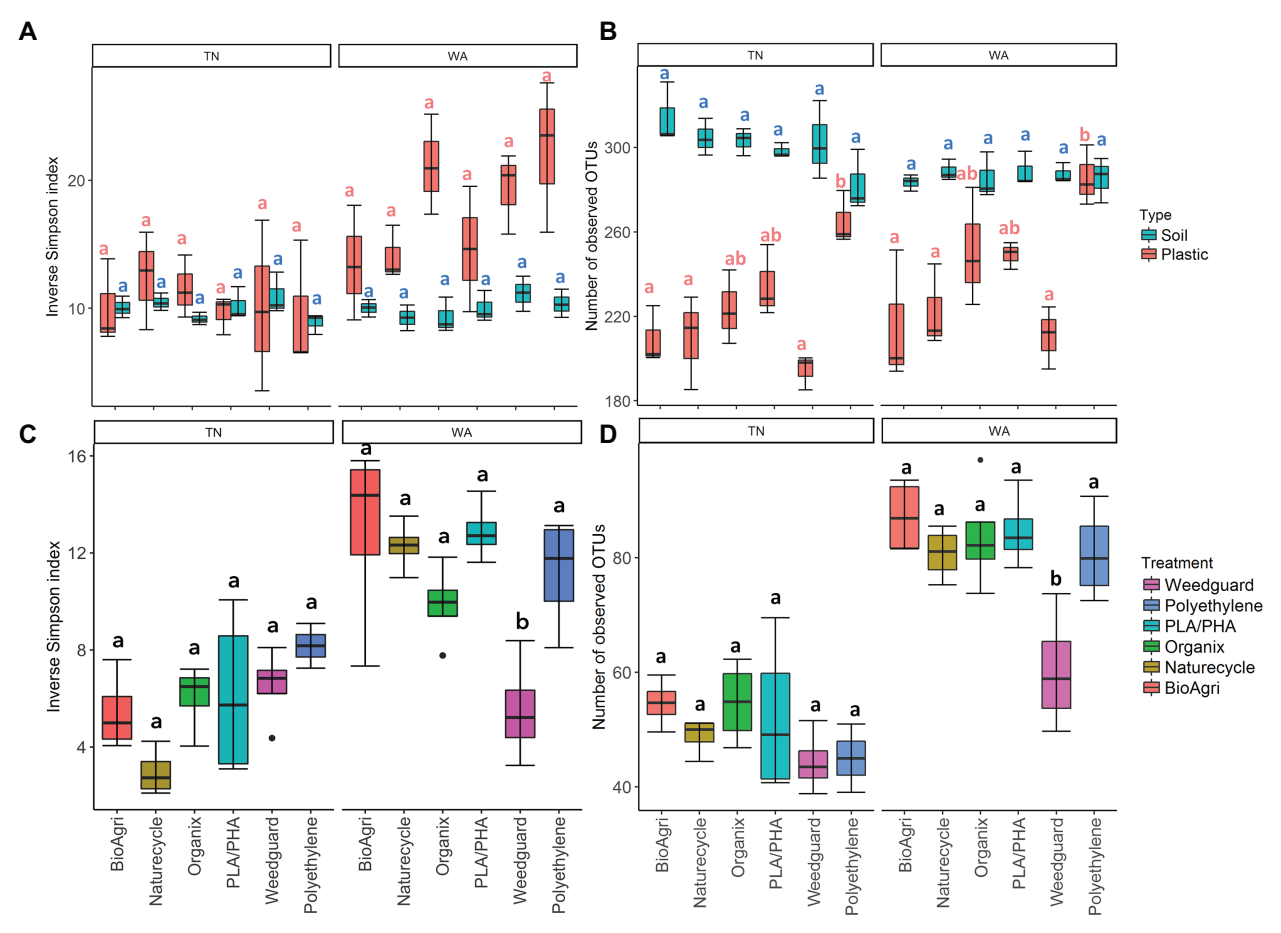
**(A)** Diversity (inverse Simpson Index) and **(B)** richness (number of observed OTUs) of bacterial communities on agriculturally-weathered BDMs and bulk soil in TN and WA. **(C)** Diversity (inverse Simpson Index) and **(D)** richness (number of observed OTUs) of eukaryotic communities on agriculturally-weathered BDMs in TN and WA. The lower and upper hinges of the boxplots correspond to the 25th and 75th percentile and the middle of the box denotes the median at 50th percentile. Whiskers denote 1.5 times the inter-quartile range. Letters indicate Tukey *post hoc* results following a one-way ANOVA. All significances tested at *α* = 0.05.

Mixed model ANOVAs showed significant interaction effects between treatment, soil/plastic type and location for gene abundance ratios, bacterial richness, and diversity estimates (Supplementary Table S15). Due to significant interaction effects, simple main effects were evaluated using one-way ANOVAs (Supplementary Tables S16, S17). Locational differences were observed for gene abundance ratios, and alpha and beta diverity estimates. Weathered plastics from TN had significantly greater 16S/ITS ratios but lower bacterial diversity compared to those from WA (Figure 4; Supplementary Figure S8; Supplementary Tables S16–S18). In contrast, WA plastics had higher eukaryotic richness and diversity compared to TN (Figure 4; Supplementary Tables S19, S20). Rarefaction curves for plastic-associated bacterial and eukaryotic communities in TN and WA are included in Supplementary Figures S9, S10.

**Figure 5:**
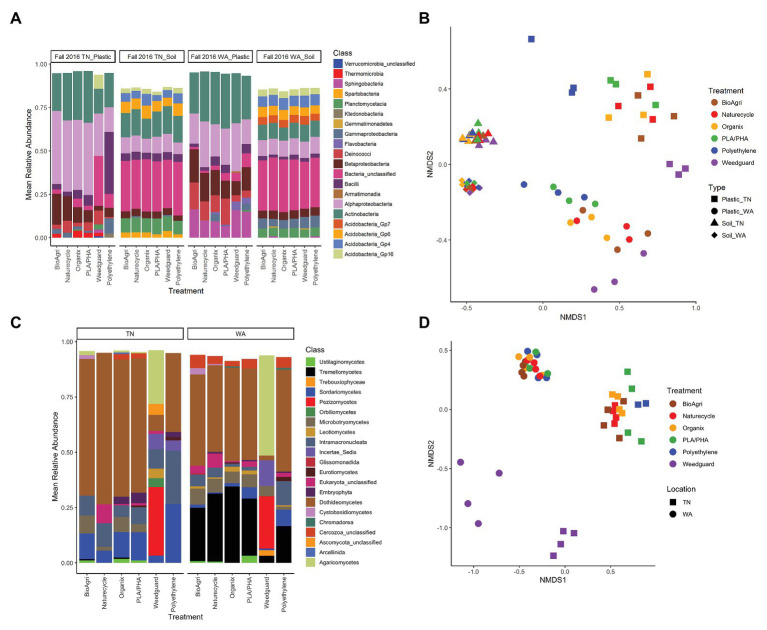
**(A)** Bacterial taxa distribution (class level) on agriculturally-weathered plastics and bulk soil (Fall 2016) for TN and WA. Mean relative abundances above a cut-off level of 2% are indicated. “Bacteria unclassified” denote taxa with relative abundance above the cut-off level of 2%, but that could not be classified. **(B)** NMDS ordination of bacterial communities on agriculturally-weathered BDMs and bulk soil in TN and WA (Fall 2016). NMDS stress value: 0.12. **(C)** Eukaryotic taxa distribution (class level) on agriculturally-weathered plastics (Fall 2016) for TN and WA. Relative abundances above a cut-off level of 2% are indicated. “Eukaryota_unclassified” denote taxa with relative abundance above the cut-off level of 2%, but that could not be classified. Phylum level classification for class incertae_sedis was *Mucoromycota* for polyethylene, and *Chytridiomycota* for Weedguard. **(D)** NMDS ordination of eukaryotic communities on agriculturally-weathered BDMs in TN and WA (Fall 2016). NMDS stress value: 0.18.

Both bacterial and eukaryotic community composition on agriculturally-weathered plastics significantly differed between TN and WA as determined by PERMANOVA (Figure 5; Supplementary Table S10). Bacterial taxa, including *Methylobacterium*, *Deinococcus*, *Hymenobacter*, *Bacilli*, *Sphingomonas*, and unclassified *Comamonadaceae* cumulatively contributed to 40% of the variation between plastic-associated communities in TN and WA. The eukaryotic taxa *Cladosporium*, *Vishniacozyma*, *Cladosproriaceae*, *Agaricomycetes*, *Filobasidium*, *Peziza*, and *Conthreep*, cumulatively contributed to about 55% of the variation between plastic-associated communities in TN and WA.

Plastic-associated communities were signifcantly different from bulk soil communities in terms of microbial gene abundances, alpha and beta diversity estimates, and community composition. 16S/ITS ratios were significantly lower (i.e., higher proportion of fungi) on agriculturally-weathered plastics compared to bulk soil in both locations (*p* < 0.001; Supplementary Table S16), with mean ratios on plastics of 1.01 ± 0.01 and 0.94 ± 0.011 for TN and WA, respectively, compared to soil ratios of 13 ± 0.01 and 1.10 ± 0.01 (Supplementary Figure S8). Plastic also had significantly lower bacterial richness (number of OTUs) compared to soil in both locations (Figure 4; Supplementary Figure S9). Interestingly, in WA only, there was a significantly higher bacterial diversity on agriculturally-weathered plastics compared to soil (Supplementary Table S17). The plastic-associated bacterial community composition was significantly different from the soil communities in both locations (*p* = 0.001; Figure 5; Supplementary Table S10). Bacterial taxa enriched on agriculturally-weathered plastics (Figure 5A) included *Actinobacteria*, *Alphaproteobacteria*, and *Deinococci*. In TN, we also observed enrichment of *Methylobacterium*, *Bacillus*, *Sphingomonas*, *Arthrobacter*, and *Deinococcus* (Supplementary Table S21). In WA, plastic communities were enriched in bacteria belonging to genera *Deinococcus*, *Hymenobacter*, *Sphingomonas*, *Methylobacterium*, *Arthrobacter*, and unclassified *Comamonadaceae*.

Comparing the different mulch treatments, we observed contrasting effects on the bacterial and eukaryotic communities: bacterial communities were altered in terms of richness, while eukaryotic communities were affected in terms of diversity and community composition. In WA, PLA/PHA had significantly higher 16S/ITS ratios compared to Organix and Weedguard (Supplementary Table S16; Supplementary Figure S8), but no abundance differences were observed between mulch treatments in TN (Supplementary Table S16; Supplementary Figure S8). Bacterial richness (bacterial OTUs) was significanty greater on PE compared to BioAgri, Naturecycle, and Weedguard treatments (Supplementary Table S17; Figure 4); however, bacterial diversity (inverse Simpson indices) was unchanged between mulch treatments. Eukaryotic richness and diversity were not different between treatments in TN. However, in WA, Weedguard had significantly lower eukaryotic richness and diversity compared to the other mulches (Supplementary Table S20; Figure 4; Supplementary Figure S10). Despite the fact that the bulk soil bacterial community composition did not differ between mulch treatments for TN or WA (*p* > 0.05), the bacterial communities associated with agriculturally-weathered mulches were significantly different between mulch treatments (*p* = 0.001; Supplementary Table S10). At both locations, *Methylobacterium*, *Arthrobacter*, and *Sphingomonas* were all higher in relative abundance on BDMs compared to PE (Supplementary Figure S11). Eukaryotic community composition differed significantly between mulch treatments (*p* = 0.001) at both locations, with the differences driven by *Peziza*, *Agaricomycetes*, *Cladosporium*, *Rhodotorula*, *Verticillium*, and *Ascomycota* (Supplementary Figure S12). Weedguard (cellulose-based mulch) had greater relative abundances of *Peziza* and *Agaricomycetes* in both TN and WA. *Cladosporium* was significantly higher in abundance on BDMs and PE compared to Weedguard mulch. PE mulch also had a significantly greater relative abundance of the ciliate group *Conthreep* compared to other mulch treatments.

## Discussion

Carbon dioxide evolution, esterase activity assays, and thermogravimetric analyses of recovered mulch fragments confirmed that biodegradation of BDMs was taking place in the enrichment cultures. Higher CO_2_ evolution from 6-week incubated BDM samples compared to 3-week incubated samples demonstrate that microbes respired more mulch carbon over time. After 6 weeks of incubation, there was no significant difference between inoculated and uninoculated cultures for any treatment. We suggest this could be because the inoculated enrichments had a dose of active soil microbes, with minimal lag time in degradation, resulting in higher respiration at week 3; by week 6, the low abundance of microbes that persisted in uninoculated films may have had more time to catch up to the inoculated treatments, resulting in comparable respiration. Evidence of BDM biodegradation was further provided by TGA, showing a decrease in thermal stability of starch in BioAgri and depolymerization of PBAT in Organix. For Naturecycle, TGA revealed some evidence that starch was preferentially degraded before the polyester. Increased esterase activities at the end of the incubations also suggest polyester degradation. Degradation of PLA oligomers can be accelerated by several esterase-type enzymes (Fukuzaki et al., 1989). Some esterase enzymes can break down polyester-type polyurethanes, which release diethylene glycol and adipic acid, the latter of which is also released upon degradation of PBAT (Nakajima-Kambe et al., 1995). Although such previous studies are based on pure polymers, we could expect similar mechanisms of ester degradation for polymer blends in mulch films.

Sterilization of plastics posed a challenge for the enrichment culture in our study. We tried UV light sterilization, which is a commonly used method for surface sterilization and has been used to sterilize polymer surfaces (Bailes et al., 2013). Despite our efforts to sterilize the plastic, we observed microbial growth in our uninoculated treatments due to contaminant microbes (albeit at much lower abundances than in the inoculated treatments). This suggests that surface sterilization by UV might not be the best decontamination method and microbes could remain embedded between polymer layers where UV might not reach. The “contaminants” in our plastic mulches included *Actinobacteria*, which are morphologically diverse bacteria ranging from coccoid, fragmenting hyphal forms to those with a highly differentiated branched mycelium. Many *Actinobacteria* also produce external spores that are resistant to UV light and dehydration (Dineen et al., 2010; Dittmann et al., 2015), which could explain their survival in plastics we exposed to UV. *Bacilli* also appeared in high relative abundance in uninoculated samples. Various species of *Bacillus*, including *Bacillus subtilis*, are known to produce dormant spores, which are 5–50 times more resistant to UV radiation than growing cells (Setlow, 2001). In our experiment, shaking the culture bottles at 30°C might have released microbes trapped between polymer layers that were protected from UV during sterilization. Even though there is a possibility of alteration of physicochemical properties of mulch *via* UV light, radiation is a better suited approach for sterilization of mulch materials given that the high temperatures in autoclaves would greatly alter the structural properties of the polymers. Other nonthermal sterilization options could also be explored in future studies, such as gamma irradiation or ethylene oxide gas sterilization.

In soil-inoculated enrichments, respiration and microbial abundances were higher than in uninoculated enrichments for most of the plastic treatments after the 3-week incubation. Different microbial taxa dominated inoculated and uninoculated enrichments, indicating that soil microbes had established and were respiring mulch carbon in inoculated enrichments, i.e., degradation was not solely due to the contaminant microbes in the plastics. Confocal microscopic images show both live and dead bacterial cells on inoculated BDMs suggesting possible biofilm formation on BDM surface by soil microbes (Bayles, 2007). Both richness and diversity indices were significantly higher for inoculated compared to uninoculated enrichments for 3- and 6-week incubations. Furthermore, isolates from inoculated culture bottles included taxa that appeared in the sequencing libraries, such as *Pseudomonas* spp., suggesting that the strains likely originated from the soil and that soil microbes were outcompeting contaminant microbes. Taken together, these results suggest that when soil inoculum is added, there appears to be successful colonization of microbes on BDMs by specific taxa. In particular, higher relative abundances of *Sphingobacteria*, *Gammaproteobacteria*, *Alphaproteobacteria*, and *Betaproteobacteria* on inoculated BDMs compared to uninoculated samples indicate a successful enrichment of these soil taxa.

Differences in bacterial communities between bulk soil and agriculturally-weathered BDMs indicated a preferential enrichment of specific taxa on plastics during field trials. We observed enrichment of distinct communities on plastics in both TN and WA; however, the communities were not identical between locations, indicating that influence of different climates and soils could have resulted in the selection of different communities (Bandopadhyay et al., 2020). The preferential enrichment of fungi on agriculturally-weathered plastics compared to bulk soil is supported by previous literature reporting fungal isolates obtained from buried mulch pieces (Moore-Kucera et al., 2014). Most of the previously cultured fungal isolates from BDMs belonged to the family *Trichocomaceae* (Moore-Kucera et al., 2014). In our field study, we found *Trichocomaceae* in the eukaryotic libraries; however, they were not dominant in terms of relative abundances, and did not differ in abundance between treatments and locations. Some field studies have shown fungal enrichment in soil after BDM incorporation (Rychter et al., 2006; Li et al., 2014b; Ma et al., 2016; Muroi et al., 2016), while others show bacterial enrichment (Li et al., 2014b). This highlights the reality that cultured degradative organisms may not always be the most dominant in communities, and justifies the importance of combining culture-dependent and culture-independent approaches to understand their ecology and importance.

Composition of the BDMs drove some of the differences in microbial colonization in lab enrichment cultures. Dense colony formation on Naturecycle plastic (composed of a co-polyester and starch) was seen in confocal images, suggesting that Naturecycle could be more amenable to colonization and/or degradation compared to other BDMs exposed to similar controlled conditions. Lower diversity estimates for Naturecycle coupled with the high CO_2_ evolved supports this argument. In general, starch-containing BioAgri and Naturecycle had similar enriched taxa, whereas PLA-containing Organix and PLA/PHA experimental films had more similar colonizers. This is expected as microbes using starch employ different mechanisms than those that can utilize PLA. In our study, *Flavobacteria* and *Sphingobacteria* had higher relative abundance on starch-containing films Naturecycle and BioAgri compared to the PLA-containing films, Organix, and PLA/PHA. Previous literature reports *β*-glucuronidase activity and starch hydrolyzing capabilities of *Flavobacteria* (Petzel and Hartman, 1986) with highly specialized abilities in using oligo and polysaccharides at very low concentrations (van Der Kooij and Hijnen, 1983). However, it is to be emphasized that although Organix contains PLA, the content is much lower than that of PLA/PHA (Supplementary Table S1). Furthermore, the major component (~80%) shared among the BDMs BioAgri, Naturecycle, and Organix is PBAT. Other studies have reported colonization of PBAT film surfaces by *Proteobacteria* such as *Hyphomicrobium* and *Caenimonas* (Muroi et al., 2016), and *Proteobacteria* was a dominant phylum across all BDMs used in the enrichment cultures. This suggests that there could be potential polyester-degrading members from *Proteobacteria* colonizing the BDMs. The similar micobial community compositions of BioAgri and Naturecycle, which was different from Organix and PLA/PHA, likely suggest a preference for similar mulch components irrespective of total percent composition.

Several bacterial and eukaryotic taxa were also seen to be preferentially enriched on agriculturally-weathered BDMs compared to PE plastic. The bacterial taxa enriched on agriculturally-weathered BDMs included *Methylobacterium*, *Arthrobacter*, and *Sphingomonas*. This suggests that microbes belonging to these genera could be potential BDM degraders. Bacterial richness was significantly lower on BDMs compared to PE. This would be expected if the BDMs are colonized by specific microbial degraders, which may be using mulch carbon and outcompeting others. PE, with a non-biodegradable and more hydrophobic surface, would likely host a wider range of microbes with no apparent selection; random colonization might explain the higher microbial richness on PE. Eukaryotic communities captured on agriculturally-weathered BDMs revealed significantly different communities on Weedguard (cellulosic) mulch compared to BDMs and PE. *Agaricomycetes* (division *Basidiomycota*) were found to be predominant on Weedguard mulch. Basidiomycetous fungi are some of the most potent degraders of cellulose growing on dead wood or litter. For the degradation of cellulose, *Basidiomycetes* utilize a set of hydrolytic enzymes typically composed of endoglucanase, cellobiohydrolase, and *β*-glucosidase. *Pezizomycetes* (class *Ascomycota*) was also significantly higher in abundance on Weedguard compared to other mulches. Cellobiose dehydrogenase is an extracellular enzyme produced by both *Ascomycetes* and *Basidiomycetes*, which efficiently oxidizes cellobiose and degrades not only cellulose but also xylan and lignin in the presence of H_2_O_2_ and chelated Fe ions (Henriksson et al., 1995; Baldrian and Valaskova, 2008). The distinct eukaryotic community seen on Weedguard mulch compared to other mulches, in both TN and WA, would be expected given the wide array of fungi that preferentially degrade cellulose compared to starch and polyester compounds. On the BDMs there was a dominance of *Dothideomycetes* and *Sordariomycetes* in TN (both division *Ascomycota*); *Dothideomycetes* and *Tremellomycetes* (division *Basidiomycota*) dominated BDMs in WA. Interestingly, studies have reported the presence of the ciliate group *Conthreep* on PE terephthalate plastics (Oberbeckmann et al., 2016) as well as PE microplastics (Kettner et al., 2019), indicating the need to explore possible depolymerization mechanisms in other soil eukaryotes.

Synthesizing the results from lab and field enrichment studies, we see several common bacterial classes that were dominant colonizers across the different BDMs. For example, in the lab enrichments we see that *Alphaproteobacteria*, *Betaproteobacteria*, *Gammaproteobacteria*, *Actinobacteria*, and *Sphingobacteria* all dominated BDMs inoculated with soil. All these classes of bacteria, with the exception of *Gammaproteobacteria*, were also dominant members on agriculturally-weathered mulch films in both TN and WA, with *Sphingobacteria* dominant on BDMs in WA. This suggests comparable results and patterns across the lab and field enrichment conditions, which allow us to focus in on the most common members of the biodegradable mulch plastisphere.

One of the limitations of the present work was that the taxonomic resolution of 16S rRNA V4 libraries cannot provide species level information. Current advances in long read technology such as PacBio circular consensus sequencing (CCS) technology to sequence full length bacterial 16S rRNA gene holds promise for high fidelity species level identification for higher taxonomic resolution. Long read sequencing with full-length 16S sequencing will also provide functional insights with improved metagenome assembled genomes eventually paving way for functional characterization of plastic-associated microbial communities.

Taken together, our results identified dominant soil microbial taxa that colonize BDMs in both lab cultures and in the field. A distinct bacterial community was observed enriched on BDMs compared to PE films in the field study, identifying potential BDM-degrading taxa. One of the unique aspects of our study a focus on films of mixed composition, rather than pure polymers, that were more realistic to plastics microbes encounter in the field. Focusing on mixed films should ultimately give us better insight into colonization, succession, and degradation patterns happening in agroecosystem soils. Finding that starch was preferentially degraded from the films was not surprising, as it is purposefully included in mulch film formulations to encourage biodegradation. The enrichment cultures demonstrated that different microbes colonized these plastics based on the composition of the plastic, with starch-containing plastics selecting for communities distinct from PLA-containing plastics. This work lays a foundation for future research focusing on characterizing microbial degradation of biodegradable plastics.

## Data Availability Statement

The datasets presented in this study can be found in online repositories. The names of the repository/repositories and accession number(s) can be found at: https://www.ncbi.nlm.nih.gov/, PRJNA631499 and https://www.ncbi.nlm.nih.gov/, PRJNA564156.

## Author Contributions

SB and JMD conceived and designed the experiments and wrote the paper with inputs from all other authors. SB, JL, KH, and MA performed the experiments. SB analyzed the data. All authors contributed to the article and approved the submitted version.

### Conflict of Interest

The authors declare that the research was conducted in the absence of any commercial or financial relationships that could be construed as a potential conflict of interest.

## Abbreviations

BDM: Biodegradable mulch
PE: Polyethylene
TN: Tennessee
WA: Washington
PLA: Polylactic acid
PHA: Polyhydroxyalkanoate
PBAT: Poly(butylene-adipate-co-terephthalate)
