# Supplementary material for: Soil Microbial Communities Associated With Biodegradable Plastic Mulch Films
Source: Front Microbiol. 2020 Nov 13;11:587074. doi: 10.3389/fmicb.2020.587074 (PMC7691482; doi:10.3389/fmicb.2020.587074)
Supplement: Supplementary file 1 [file Data_Sheet_1.pdf]

Supplementary Figures and Tables

Supplementary Figures

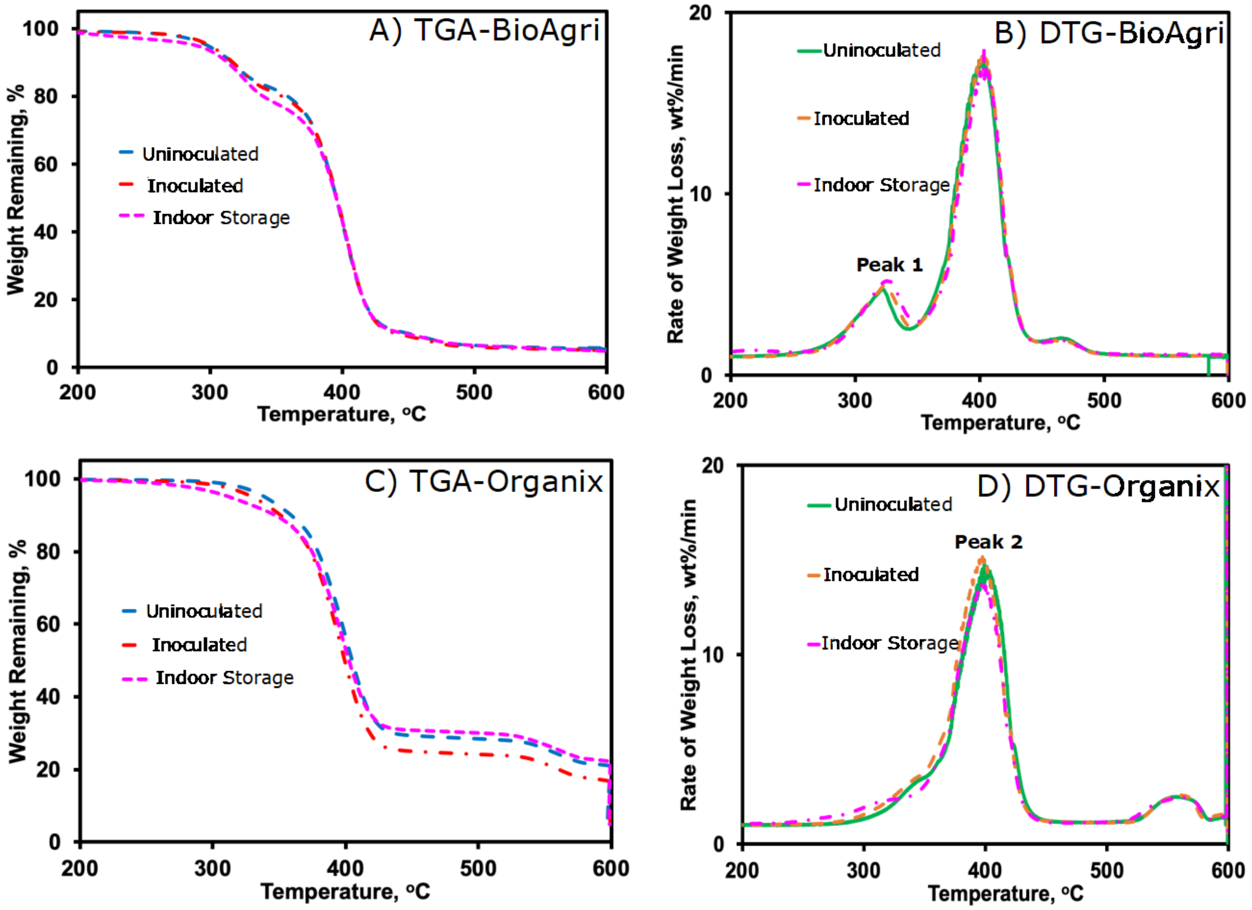

Figure S1: Thermograms (TGA) (A and C) and differential thermograms (DTG) (B and D) of BioAgri and Organix from lab incubation study showing percent weight remaining and rate of weight loss after heating the plastics at a constant rate (10°C/min). "Indoor Storage" refers to unweathered mulch films which were kept in a storage cabinet in the dark at room temperature. Peak 1 is attributable to starch in BioAgri, Peak 2 is attributable to PBAT in Organix and BioAgri.

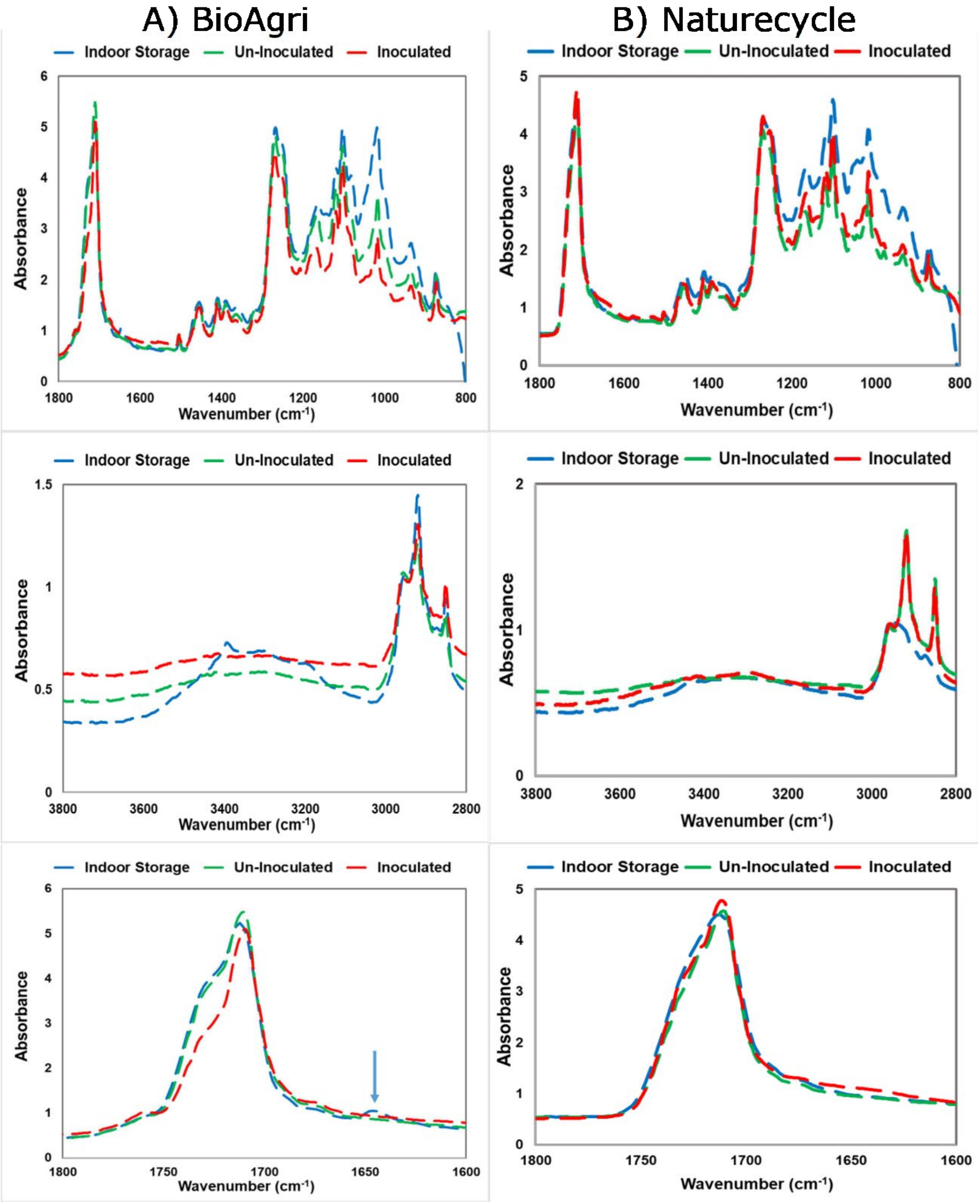
A) BioAgri
B) Naturecycle
Indoor Storage
Un-Inoculated
Inoculated
Absorbance
Wavenumber (cm-1)
1800
1600
1400
1200
1000
800
3800
3600
3400
3200
3000
2800
1750
1700
1650
1600

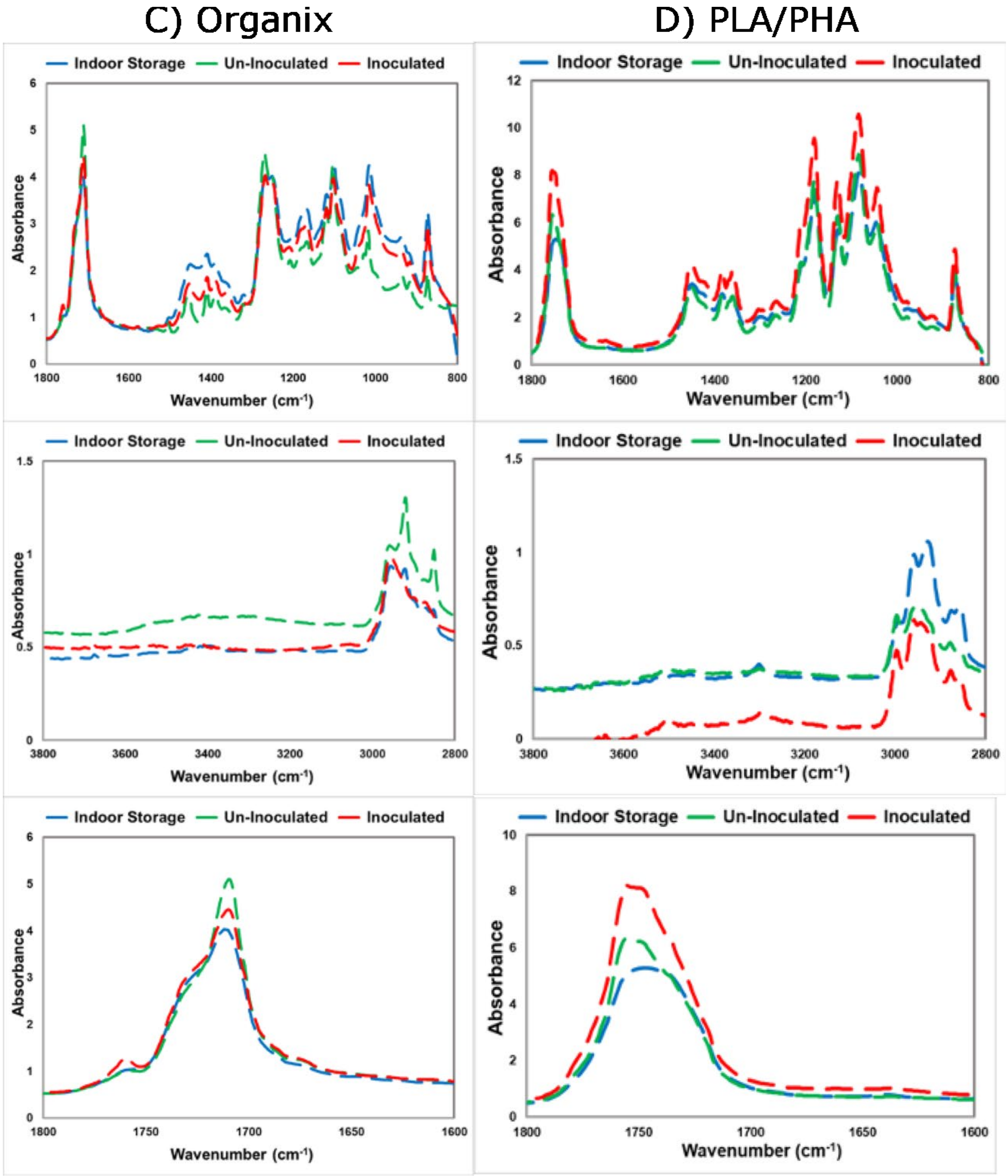

Figure S2: Fourier Transform Infra-red (FTIR) spectra of A) BioAgri, B) Naturecycle mulch, C) Organix, D) PLA/PHA films from lab incubation study at three conditions: indoor storage, un-inoculated and inoculated.

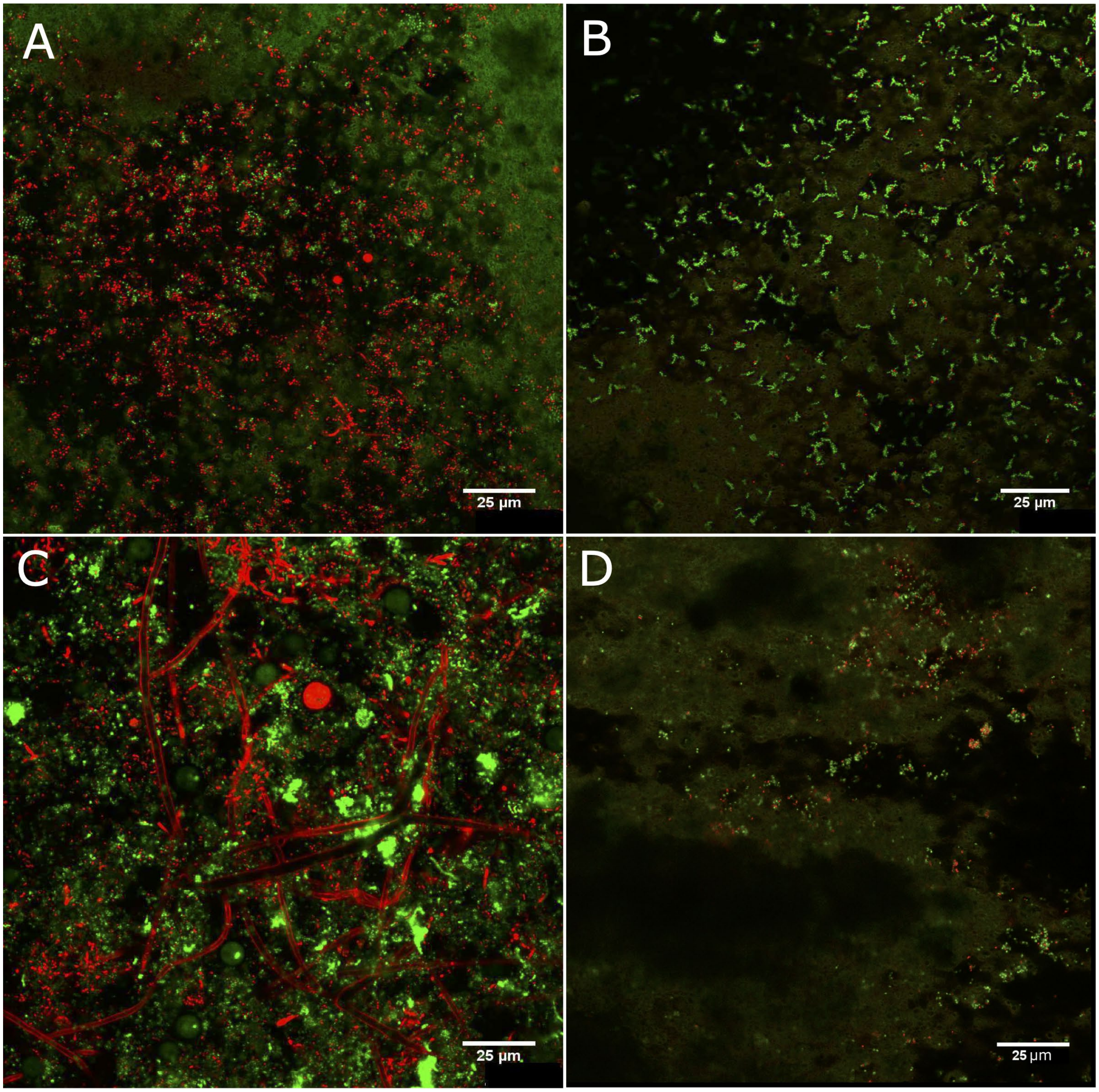

Figure S3: Confocal images of BDMs after enrichment using a Leica™ white light laser confocal system. a) BioAgri inoculated (3 weeks), b) BioAgri uninoculated (3 weeks), c) Naturecycle inoculated (3 weeks), d) Naturecycle uninoculated (6 weeks). Staining was done using a LIVE/DEAD® BacLight™ Bacterial Viability Kit. Green: live cells, Red: dead cells. Scale bars=25 µm.

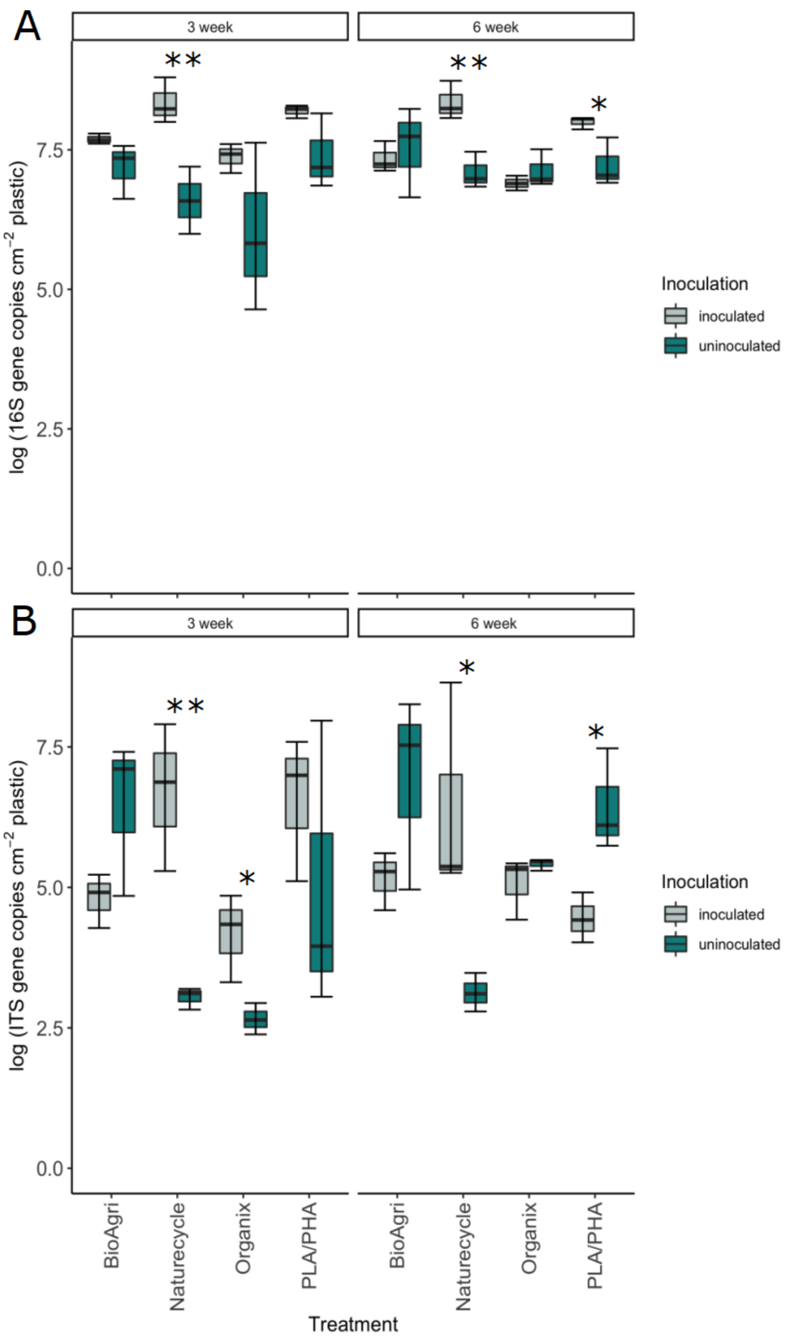

Figure S4: a) 16S rRNA (bacterial) and b) ITS (fungal) gene abundances for 3-week and 6-week incubations. Gene abundances are log transformed. The lower and upper hinges of the boxplots correspond to the 25th and 75th percentiles and the middle of the box denotes the median at 50th percentile. Whiskers show 1.5 times the inter-quartile range. Asterisks denote significant differences between uninoculated and inoculated enrichments from a one-way ANOVA; $*p \leq 0.05$, $**p \leq 0.01$, $***p \leq 0.001$.

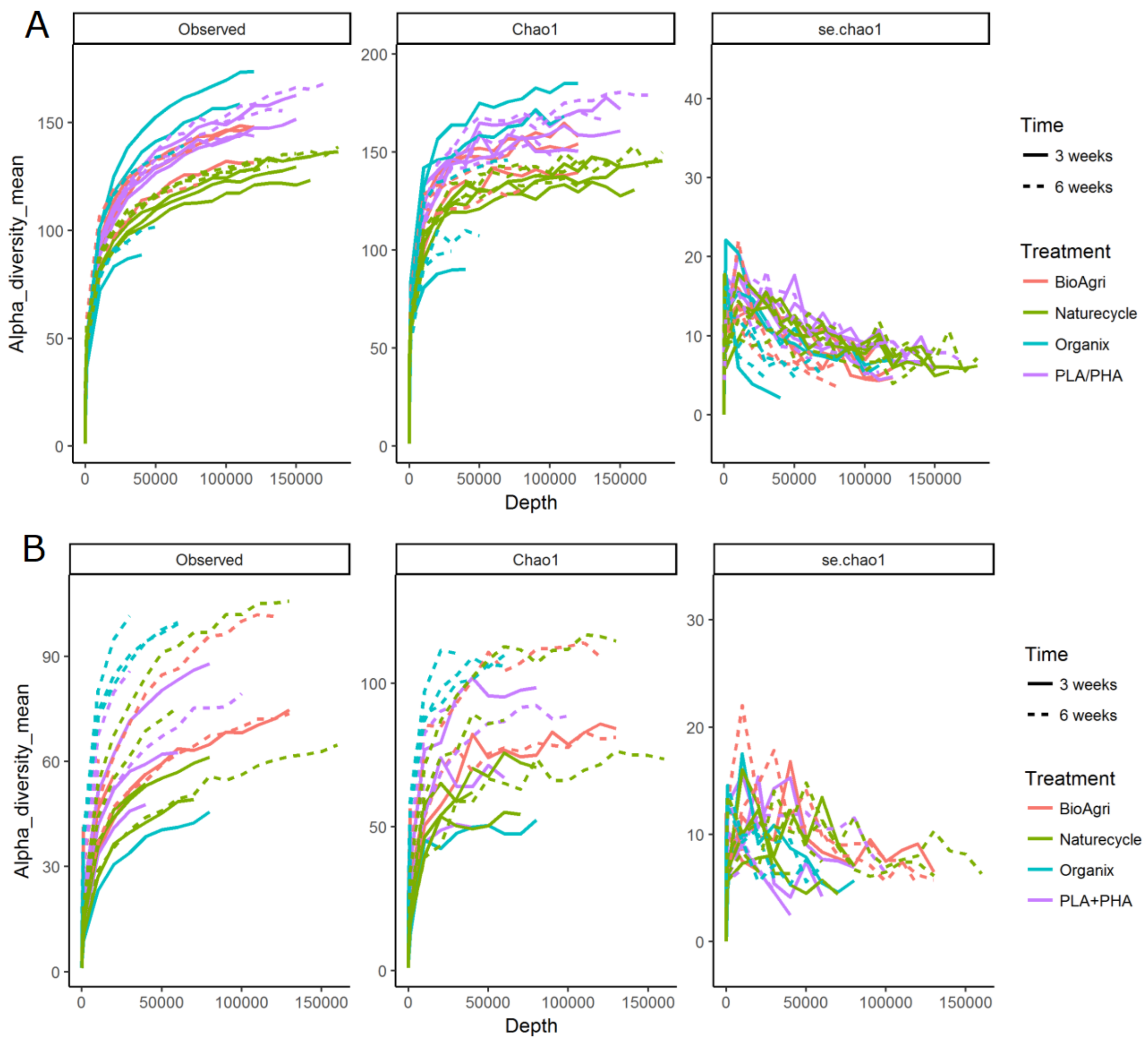

Figure S5: Alpha diversity measures calculated for lab enriched bacterial communities in a) Inoculated and b) Uninoculated samples. All reads were scaled to even depth. Observed: Observed number of OTUs. se: standard error.

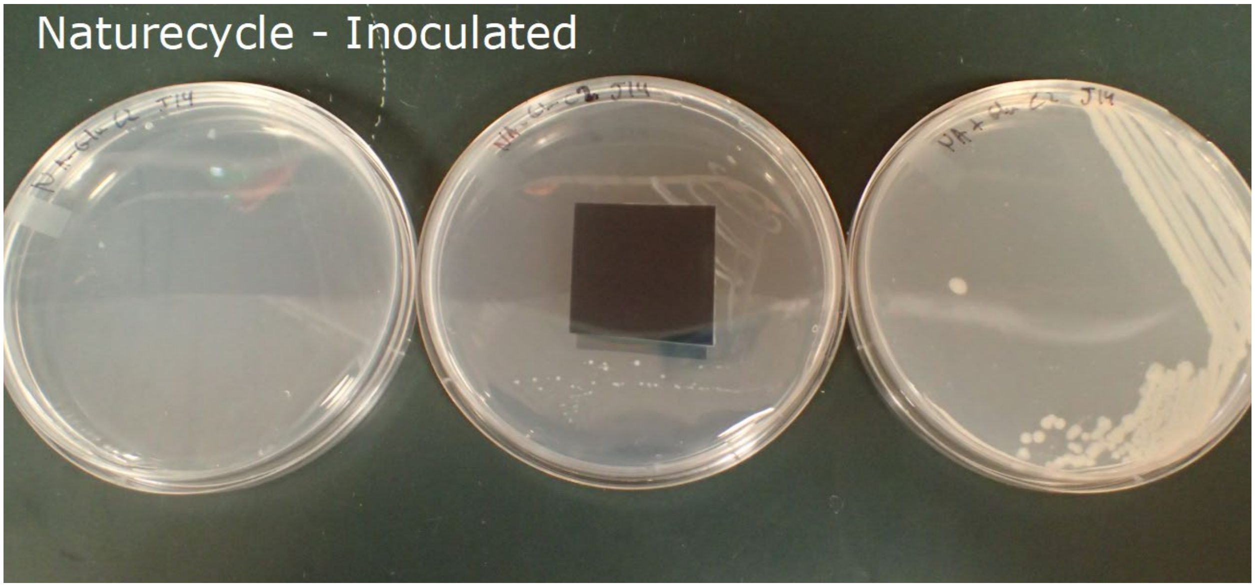

Figure S6: Images of example plates used for the isolation of BDM degrading microbes. Plate contained M9 minimal agar with plastic as sole carbon source. Streaking was completed using colonies from spread plates made with inoculum from culture bottles incubated for six weeks. Picture shows (left to right) negative control (no plastic, no glucose), treatment (media with plastic, no glucose), positive control (media with glucose).

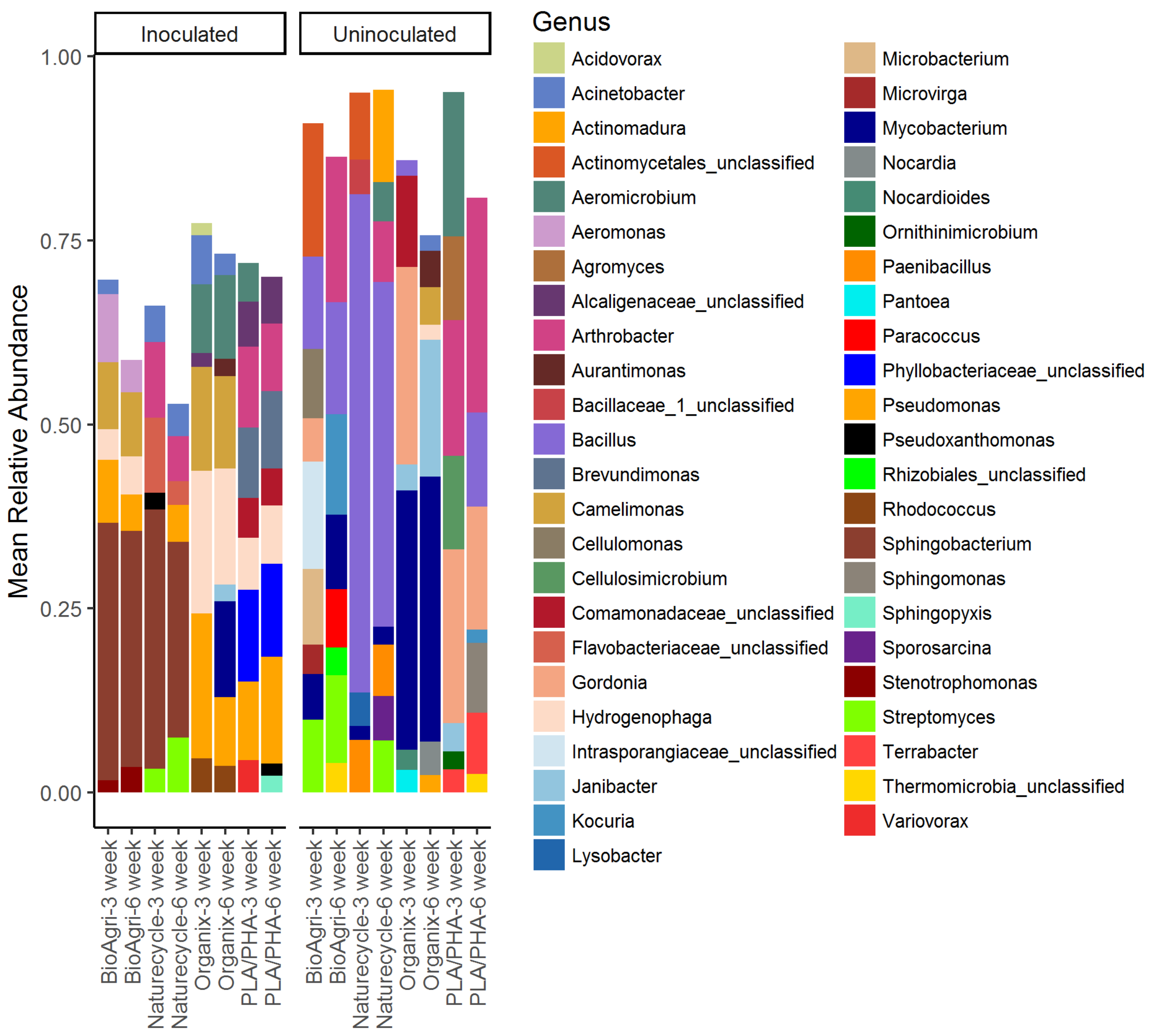

Figure S7: Bacterial taxa distribution (genus level) on mulch treatments for the enrichment cultures conducted for 3 weeks and 6 weeks. Mean relative abundances above a cut-off level of 5% are indicated. “unclassified" denote taxa with relative abundance above the cut-off level of 5%, but that could not be classified.

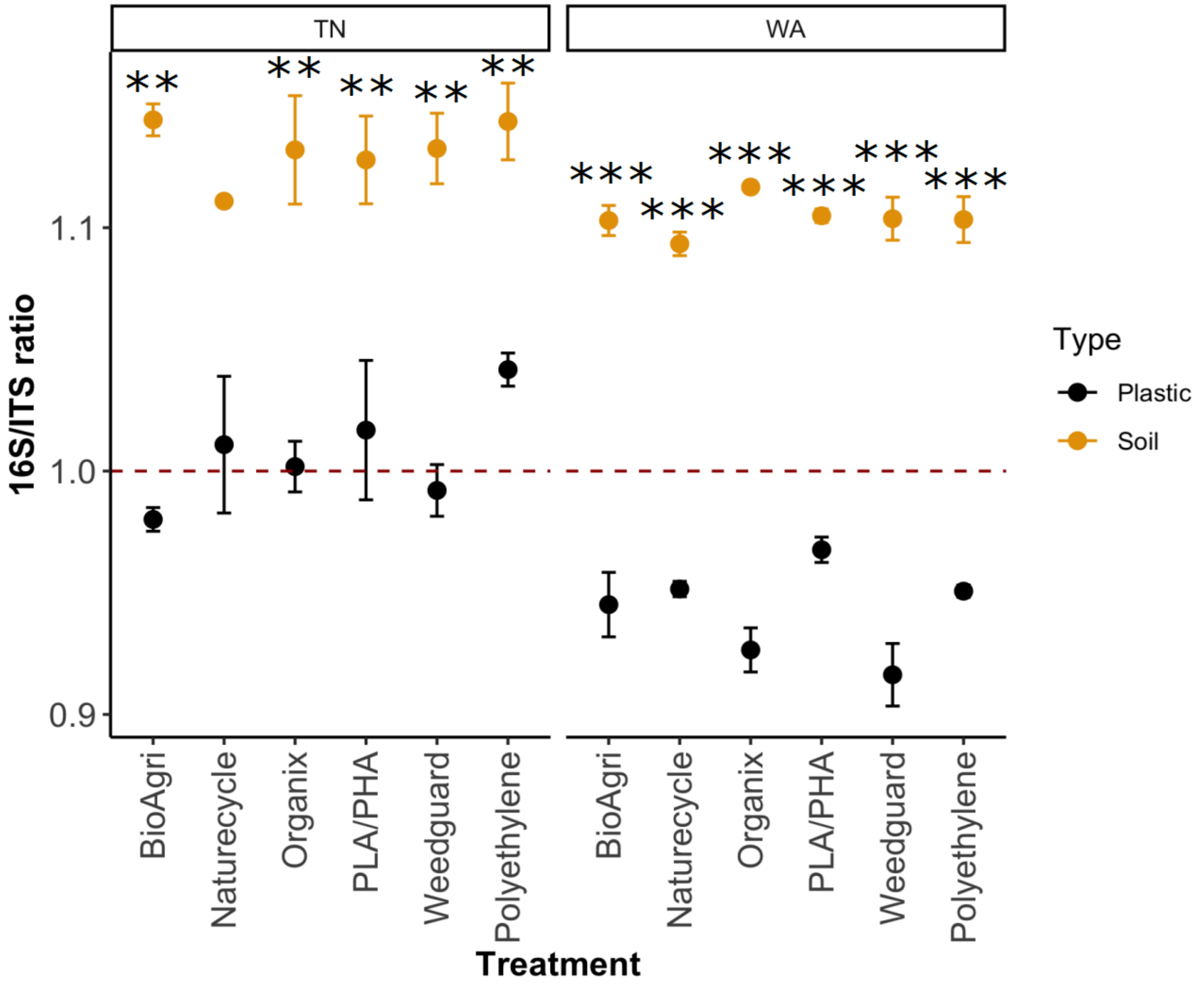

Figure S8: 16S/ITS gene abundance ratios for agriculturally-weathered plastics and bulk soil. Error bars indicate the standard error of the mean (n = 4 for all treatments, except for Naturecycle soil and plastic treatments in TN and WA and BioAgri soil and plastic treatments in TN, where n = 3). Red dashed line denotes a 1:1 threshold. Asterisks denote significant differences in 16S/ITS ratios between soil and plastic treatments from a t-test; *$p \leq 0.05$, **$p \leq 0.01$, ***$p \leq 0.001$.

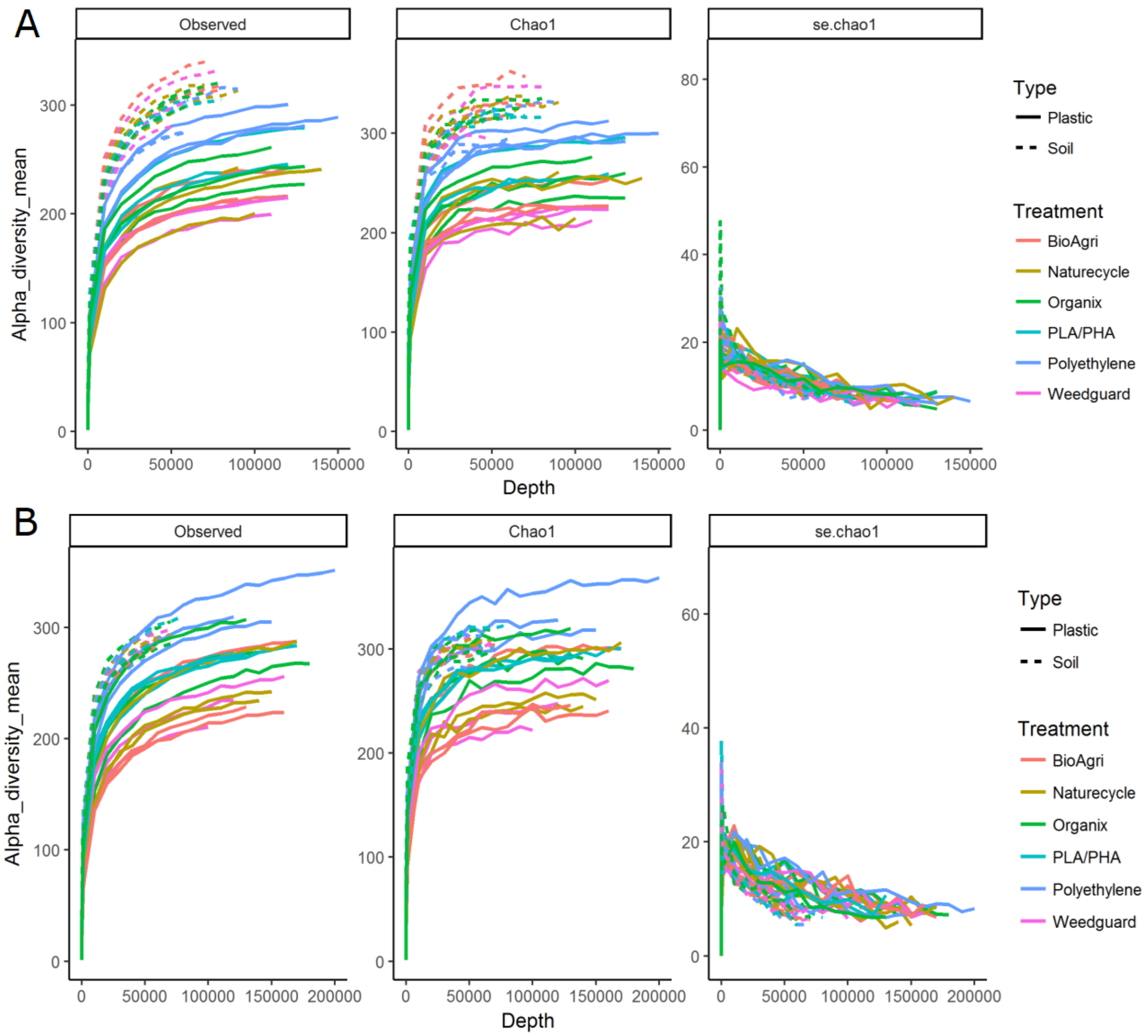

Figure S9: Richness measures as a function of sampling depth calculated for agriculturally-weathered plastic-associated bacterial communities and soil communities in a) TN and b) WA. All reads were scaled to even depth. Observed: Observed number of OTUs. se: standard error.

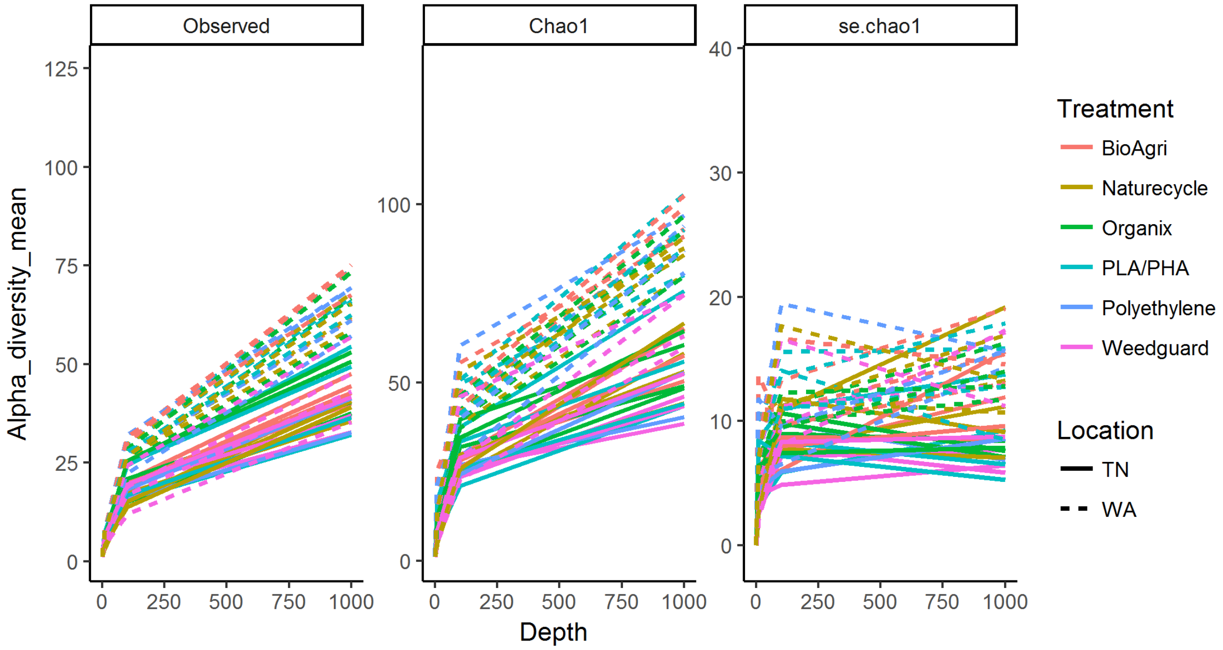

Figure S10: Richness measures as a function of sampling depth calculated for agriculturally-weathered plastic-associated eukaryotic communities in TN and WA. All reads were scaled to even depth. Observed: Observed number of OTUs. se: standard error.

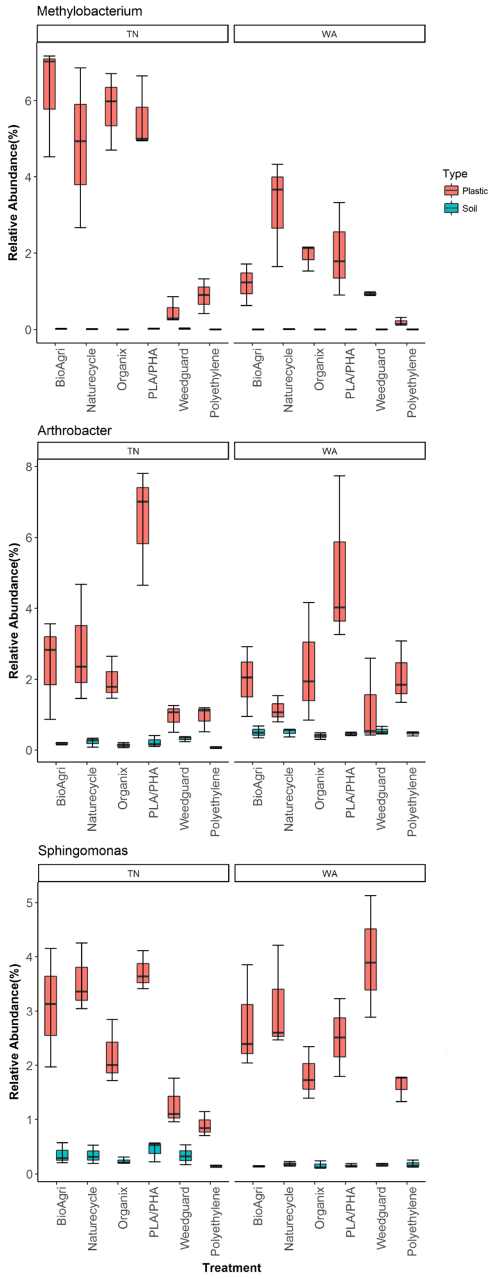

Figure S11: Percent relative abundance of taxa (Genus level) driving variance in microbial communities between soils and plastics in TN and WA as determined via SIMPER analysis. The lower and upper hinges of the boxplots correspond to the 25th and 75th percentiles and the middle of the box denotes the median at 50th percentile. Whiskers denote 1.5 times the inter-quartile range. Differences between plastic treatments were evaluated using Kruskal-Wallis non-parametric test. Significant differences were observed between plastic treatments in TN for *Methylobacterium* ($p = 0.03$), *Arthrobacter* ($p = 0.03$), and *Sphingomonas* ($p = 0.02$), and in WA significant differences were seen between plastic treatments for *Methylobacterium* ($p = 0.03$) and *Sphingomonas* ($p = 0.03$). Significance was set at $\alpha < 0.05$.

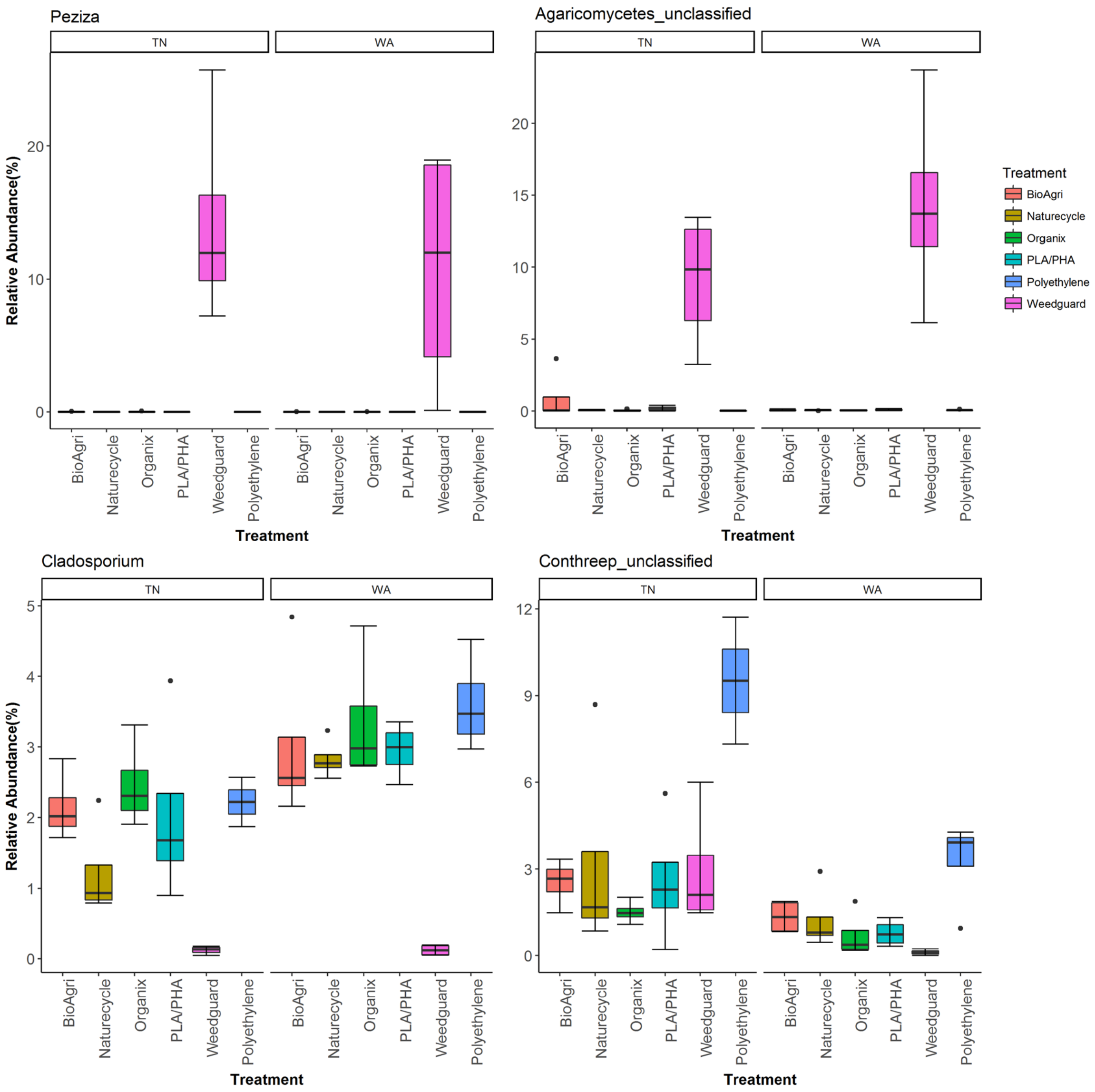

Figure S12: Percent relative abundance of taxa (genus level) driving overall differences in eukaryotic communities between agriculturally-weathered plastics in TN and WA as obtained via SIMPER analysis. The lower and upper hinges of the boxplots correspond to the 25th and 75th percentiles and the middle of the box denotes the median at 50th percentile. Lower whisker equals the smallest observation greater than or equal to lower hinge minus 1.5 times the inter-quartile range, i.e. the distance between the first and third quartiles. Upper whisker equals largest observation less than or equal to upper hinge plus 1.5 times the inter-quartile range, i.e. the distance between the first and third quartiles. “Taxa_unclassified” denote taxa which could not be classified at the genus level. Differences between plastic treatments were evaluated using Kruskal-Wallis non-parametric test. Significant differences were observed between plastic treatments in TN for *Peziza* ($p = 0.007$) and *Cladosporium* ($p = 0.02$). In WA significant differences were seen between plastic treatments for *Peziza* ($p = 0.01$), *Agaricomycetes_unclassified* ($p = 0.03$), *Cladosporium* ($p = 0.03$) and *Conthreep_unclassified* ($p = 0.01$). Significance was set at $\alpha < 0.05$.

Supplementary Tables

Table S1: Manufacturers, major constituents, and physicochemical properties of the mulches used in the study. Biobased content data was provided by the manufacturers. Data reported from Hayes et al. (2017).

Mulches	Manufacturer	Major constituents	Weight ($g\ m^{-2}$)	Thickness (µm)	Elongation (%)	Contact angle (º)	Total carbon (%)	Biobased content (%)
BioAgri®	BioBag Americas, Inc., Dunedin, FL	Mater-Bi® grade EF04P (blend of starch and PBAT)	18.0	26	260	87.6	57.6	20-25
Naturecycle	Custom Bioplastics, Burlington, WA	Blend of starch and polyesters	25.4	48	213	69.2	54.8	˜ 20
Organix A.G. Film™	Organix Solutions, Maple Grove, MN	BASF®ecovio® grade M2351(blend of PLA and PBAT)	17.8	20	273	86.2	51.4	10-20
Experimental PLA/PHA	Metabolix Inc., Cambridge, MA	88.4% MD05-1501 (56% Ingeo PLA, 24% Mirel™ amorphous PHA, 15% $CaCO_3$ and 5% plasticizer and processing additives), 10.0% Techmer PLA M91432 (20% carbon black in PLA 3052) and 1.6% PLA	25.0	33	247	67.8	47.5	86

Mulches	Manufacturer	Major constituents	Weight ($g\ m^{-2}$)	Thickness (µm)	Elongation (%)	Contact angle (º)	Total carbon (%)	Biobased content (%)
WeedGuardPlus®	Sunshine Paper Co., Aurora, CO	Cellulose	240	479	6.4	<10	46.0	100
Polyethylene	Filmtech, Allentown, PA	Linear low-density polyethylene	25.4	47	578	79.3	82.9	< 1

PBAT: Polybutylene co-adipate co-terephthalate; PLA: Polylactic acid; PHA: Poly(hydroxyalkanoate)

Table S2: Environmental data collected in Fall 2016 when plastic mulches and soil were collected from the field site in TN and WA. Mean values are reported with standard errors. Full soil data is reported in Sintim et al. (2019).

Location	Bulk density (g cm^{-3})	Soil texture	Soil type	pH	Mean annual soil temperature (°C)	Mean annual precipitation (mm)
ETREC, Knoxville, TN	1.22 ± 0.01	Silt loam	Typic hapludult	5.8 ± 0.08	16.8 ± 0.18	46.77
NWREC, Mount Vernon, WA	1.13 ± 0.01	Silt loam	Typic fluvaquents	5.54 ± 0.03	12.54 ± 0.12	49.55

Table S3: Weight-averaged molecular weight (Mw) and polydispersity index (PDI) of plastic mulches after 6-week incubation in enrichment cultures using gel permeation chromatography. Data represents one replicate measurement from BioAgri and PLA/PHA inoculated and uninoculated treatments.

	**Mw (kDa)**	**PDI**
BioAgri uninoculated	369.76	2.90
BioAgri inoculated	332.47	2.59
PLA/PHA uninoculated	333.21	1.88
PLA/PHA inoculated	340.76	1.86

Mw: Weight-averaged Molecular weight (unit: kilodalton), PDI: polydispersity index

Table S4: Esterase enzyme activity in spent culture media after 3 weeks and 6 weeks of incubation. Activities are reported with standard errors of the mean. All technical replicates were assayed in triplicates. Bold numbers and asterisks denote significant difference in activities between 3 week and 6 week incubation for each treatment (*$p \leq 0.05$, **$p \leq 0.01$, ***$p \leq 0.001$).

Treatment (time)	Esterase enzyme activity in spent media of enrichment culture (activity expressed in µg *p*-nitrophenol released µl $^{-1}$ minute $^{-1}$)	T statistic
BioAgri (3 weeks)	1.06 ± 0.23	**-4.26***
BioAgri (6 weeks)	3.05 ± 0.31	
PLA+PHA (3 weeks)	2.14 ± 0.06	**-4.50***
PLA+PHA (6 weeks)	2.73 ± 0.13	
Organix (3 weeks)	1.80 ± 0.46	0.25
Organix (6 weeks)	1.46 ± 0.87	
Naturecycle (3 weeks)	1.59 ± 0.35	-2.13
Naturecycle (6 weeks)	2.63 ± 0.21	

Table S5: F values of three-way ANOVAs for bacterial (16S rRNA) and fungal (ITS) gene copy abundances, and 16S/ITS ratios obtained from lab incubation study (type III tests reported). Bold numbers and asterisks denote significant difference between the factor levels and interaction effects (*$p \leq 0.05$, **$p \leq 0.01$, ***$p \leq 0.001$).

		16S copies	ITS copies	16S/ITS ratios
Factor (levels)	Time (3 weeks and 6 weeks)	0.603	0.141	0.381
	Inoculation (inoculated, uninoculated)	1.291	3.03	2.352
	Treatment (BioAgri, Naturecycle, PLA/PHA, Organix)	1.995	**3.548***	1.605
Interaction effects	Time:Inoculation	1.241	0.006	0.125
	Time:Treatment	0.204	1.902	1.92
	Inoculation:Treatment	1.507	**5.305****	**4.011***
	Time:Inoculation:Treatment	1.010	1.421	1.805

Table S6: F values of one-way ANOVAs testing differences between 3 weeks and 6 weeks incubation times for 16S, ITS and 16S/ITS ratios for inoculated and uninoculated enrichments within individual treatments. Bold numbers and asterisks denote significant difference between 3-week and 6-week time points (*p ≤ 0.05, **p ≤ 0.01, ***p ≤ 0.001).

Factor (level)	Specific treatment	16S copies	ITS copies	16S/ITS ratio
Time (3 weeks and 6 weeks) for inoculated enrichments	BioAgri	4.279	0.761	2.24
	PLA/PHA	4.805	7.162	**7.541***
	Naturecycle	0	0.038	0.16
	Organix	**7.561***	2.585	2.847
Time (3 weeks and 6 weeks) for uninoculated enrichments	BioAgri	0.431	0.129	0.027
	PLA/PHA	0.141	0.82	2.171
	Naturecycle	1.621	0.127	0.028
	Organix	1.509	**257.6*****	4.565

Table S7: Mean richness (number of observed OTUs) and diversity (inverse Simpson Index) for plastic-associated bacterial communities in laboratory enrichments ± standard deviations.

Treatment	Time	Richness	Diversity Index
BioAgri-Inoculated	3 weeks	58 ± 4	6.355 ± 0.523
BioAgri-Inoculated	6 weeks	66 ± 5	7.385 ± 2.22
BioAgri-Uninoculated	3 weeks	34 ± 19	3.336 ± 0.659
BioAgri-Uninoculated	6 weeks	28 ± 8	3.982 ± 0.782
Naturecycle-Inoculated	3 weeks	59 ± 2	6.553 ± 2.923
Naturecycle-Inoculated	6 weeks	64 ± 2	8.907 ± 2.246
Naturecycle-Uninoculated	3 weeks	18 ± 4	2.137 ± 0.873
Naturecycle-Uninoculated	6 weeks	21 ± 5	3.061 ± 1.083
Organix-Inoculated	3 weeks	56 ± 8	7.447 ± 1.221
Organix-Inoculated	6 weeks	58 ± 6	10.15 ± 1.318
Organix-Uninoculated	3 weeks	30 ± 16	2.563 ± 1.142
Organix-Uninoculated	6 weeks	49 ± 2	5.279 ± 1.221
PLA+PHA-Inoculated	3 weeks	60 ± 4	12.75 ± 1.131
PLA+PHA-Inoculated	6 weeks	59 ± 1	12.15 ± 2.19
PLA+PHA-Uninoculated	3 weeks	23 ± 5	3.025 ± 1.023
PLA+PHA-Uninoculated	6 weeks	36 ± 7	3.271 ± 0.649

Table S8: F values from three-way ANOVAs for richness (number of observed OTUs) and diversity (inverse Simpson Index) estimates obtained from lab incubation study (type III tests reported). Bold numbers and asterisks denote significant difference between the factor levels and interaction effects (*p ≤ 0.05, **p ≤ 0.01, ***p ≤ 0.001).

		Richness F	Diversity Index F
Factor (levels)	Time (3 weeks and 6 weeks)	1.574	0.721
	Inoculation (inoculated, uninoculated)	**14.021*** **	**6.193***
	Treatment (BioAgri, Naturecycle, PLA/PHA, Organix)	0.133	**12.372*****
Interaction effects	Time:Inoculation	2.615	0.05
	Time:Treatment	0.365	1.528
	Inoculation:Treatment	1.589	**5.778****
	Time:Inoculation:Treatment	2.646	0.304

Table S9: F values from one-way ANOVAs testing differences in richness (number of observed OTUs) and diversity (inverse Simpson Index) in lab enrichments between mulch treatments, inoculated and uninoculated samples and time of incubation. Bold numbers and asterisks denote significant difference between the factor levels tested (*$p \leq 0.05$, **$p \leq 0.01$, ***$p \leq 0.001$).

Factor	Levels	Within	Richness F	Diversity Index F
Treatment	BioAgri, Naturecycle, Organix, PLA/PHA	3 weeks (inoculated)	0.306	**9.433****
		6 weeks (inoculated)	2.551	2.946
		3 weeks (uninoculated)	0.908	0.934
		6 weeks (uninoculated)	**11.78****	3.257
Inoculation	Inoculated, uninoculated	3 weeks	**77.34*****	**12.94***
		6 weeks	**20.21****	**26.14****
Time	3 weeks, 6 weeks	Inoculated	2.807	0.572
		Uninoculated	0.977	4.017

Table S10: PERMANOVA statistics (F values) to test for significant differences in microbial community composition. PERMANOVAs were computed with Adonis function in R vegan package. Bold numbers and asterisks indicate significant differences between the levels tested; ***p ≤ 0.001, ** p ≤ 0.01, *p ≤ 0.05.

	Factor	Levels	Within	PERMANOVA F
Lab enrichement cultures	Time	3 weeks, 6 weeks	PLA/PHA (inoculated)	0.557
			Organix (inoculated)	2.203
			Naturecyle (inoculated)	0.816
			BioAgri (inoculated)	0.911
	Treatment	BioAgri, Naturecycle, Organix, PLA/PHA	3 weeks (inoculated)	**12.454*** **
			6 week (inoculated)	**10.283*** **
	Innoculation	Innoculated, uninoculated	3 weeks	**7.636*** **
			6 weeks	**7.252*** **
Agriculturally-weathered films: Bacterial communities	Location	TN, WA	Soil	**45.458*** **
			Plastic	**9.856*** **
	Type	Soil, plastic	TN	**59.551*** **
			WA	**107.77*** **
	Treatment	BioAgri, Naturecycle, Organix, PLA/PHA	TN Soil	1.035
			WA Soil	1.133
			TN Plastic	**5.172*** **
			WA Plastic	**2.833*** **
Agriculturally-weathered films: Eukaryotic communities	Location	TN, WA		**16.666*** **
	Treatment	BioAgri, Naturecycle, Organix, PLA/PHA	Location (TN)	**6.782*** **
			Location (WA)	**10.419*** **

Table S11: Summary statistics (F values) for one-way ANOVAs testing differences between inoculated and uninoculated enrichments for 16S, ITS and 16S/ITS ratios at 3-week and 6-week time points. Bold numbers and asterisks indicate significant differences between the levels tested; ***$p \leq 0.001$, ** $p \leq 0.01$, *$p \leq 0.05$.

Factor (level)	Treatment	16S copies	ITS copies	16S/ITS ratio
Inoculation (3-week inoculated and uninoculated samples)	BioAgri	3.096	3.731	4.721
	PLA/PHA	4.078	0.871	0.996
	Naturecycle	**17.32****	**22.53****	**12.7***
	Organix	2.305	**9.901***	0.994
Inoculation (6-week inoculated and uninoculated samples)	BioAgri	0.16	2.831	6.024
	PLA/PHA	**8.781***	**11.52***	**39.39****
	Naturecycle	**20.69****	**8.559***	**11.6***
	Organix	1.139	1.218	0.398

Table S12: Identity of isolated strains from enrichment cultures. Identity determined by BLAST alignment of 16S rRNA genes amplified with 8F and 1492R primers to NCBI Genbank database.

BDM used in enrichment	Top BLAST hit	Sequence identity with NCBI Genbank
Naturecycle	*Streptomyces* sp. 1	97%
	Streptomyces sp. 2	98%
	Arthrobacter sp.	95%
	Streptomyces sp. 3	99%
	Microbacterium sp.	98%
	Streptomyces sp. 4	97%
	Variovorax sp.	99%
BioAgri	*Arthrobacter* sp.	97%
	Pseudomonas sp.	96%
	Streptomyces sp.	98%
Organix	*Devosia* sp.	93%

Table S13: F values of one-way ANOVAs testing differences between treatments for 16S, ITS and 16S/ITS ratios for 3-week and 6-week incubations (inoculated and uninoculated). Bold numbers and asterisks indicate significant differences between treatments; ***$p \leq 0.001$, ** $p \leq 0.01$, *$p \leq 0.05$.

Factor (level)	Time	16S copies	ITS copies	16S/ITS ratio
Treatment (BioAgri, Naturecycle, Organix, PLA/PHA) for inoculated enrichments	3 weeks	**9.35****	**4.504***	2.47
	6 weeks	**22.19*****	1.846	3.475
Treatment (BioAgri, Naturecycle, Organix, PLA/PHA) for uninoculated enrichments	3 weeks	1.368	**4.218***	2.55
	6 weeks	0.466	**8.619****	**20.82*****

Table S14: Tukey post hoc tests for 3-week and 6-week incubations showing pairwise comparison of 16S and ITS copy numbers between inoculated mulch treatments. Data shown for only those time points which showed significant differences by one-way ANOVA. Bold numbers and asterisks indicate significant differences between treatments; ***$p \leq 0.001$, ** $p \leq 0.01$, *$p \leq 0.05$.

16S or ITS abundance	Pairwise comparison (Tukey post hoc)	P value
16S copies after 3-week incubation (inoculated)	Naturecycle - BioAgri	0.056
	Organix - BioAgri	0.45
	PLA+PHA - BioAgri	0.15
	Organix - Naturecycle	**0.007****
	PLA+PHA - Naturecycle	0.89
	PLA+PHA - Organix	**0.02***
16S copies after 6-week incubation (inoculated)	Naturecycle - BioAgri	**0.004****
	Organix - BioAgri	0.19
	PLA+PHA - BioAgri	**0.04***
	Organix - Naturecycle	**0.0003*****
	PLA+PHA - Naturecycle	0.33
	PLA+PHA - Organix	**0.002****
ITS copies after 3-week incubation (inoculated)	Naturecycle - BioAgri	0.19
	Organix - BioAgri	0.87
	PLA+PHA - BioAgri	0.23
	Organix - Naturecycle	0.07
	PLA+PHA - Naturecycle	0.999
	PLA+PHA -Organix	0.08

Table S15: F values from a mixed model ANOVA for 16S/ITS ratios, richness (number of observed OTUs) and diversity (inverse Simpson Index) estimates obtained from field study (type III tests reported). Bold numbers and asterisks indicate significant differences between factor levels and interaction effects; ***p ≤ 0.001, ** p ≤ 0.01, *p ≤ 0.05.

		16S/ITS ratios	Richness F	Diversity Index F
Factor (levels)	Treatment (BioAgri, Naturecycle, PLA/PHA, Organix, Weedguard, Polyethylene)	1.59	**7.31*** **	0.999
	Type (Soil and Plastic)	**2704.66*** **	**355.95*** **	**33.019*** **
	Location (TN and WA)	**57.84*** **	0.03	**21.970** **
Interaction effects	Treatment:Type	**9.26*** **	**14.63*** **	1.673
	Treatment:Location	0.72	0.96	1.908
	Type:Location	**44.98*** **	**21.13*** **	**24.316*** **
	Treatment:Type:Location	**2.75* **	0.35	1.062

Table S16: F values of one-way ANOVAs testing differences in 16S/ITS ratios on agriculturally-weathered plastics between locations and treatments, and between bulk soil and agriculturally-weathered plastic. Bold numbers and asterisks indicate significant differences between factor levels tested; ***p ≤ 0.001, ** p ≤ 0.01, *p ≤ 0.05.

Factor	Levels	Within	F value
Location	TN, WA		**57.46*** **
Treatments	BioAgri, Naturecycle, PLA/PHA Organix, Polyethylene, Weedguard	TN	1.402
		WA	**4.564** **
Type	Soil, plastic	TN	**174.2*** **
		WA	**377.9*** **

Table S17: F values from one-way ANOVAs testing differences in richness (number of observed OTUs) and diversity (inverse Simpson Index) metrics for field enrichment study by mulch treatments, location and soil and plastic communities. Bold numbers and asterisks indicate significant differences between factor levels tested; ***p ≤ 0.001, ** p ≤ 0.01, *p ≤ 0.05.

Factor	Levels	Within	Richness F	Diversity Index F
Treatment	BioAgri, Naturecycle, Organix, PLA/PHA	Plastic (TN)	**7.374****	0.239
		Soil (TN)	2.204	1.755
		Plastic (WA)	**5.567****	2.538
		Soil (WA)	0.226	1.133
Location	TN, WA	Plastic	1.105	**17.41****
		Soil	**10.84****	0.017
Type	Soil, plastic	TN	**50.36 *****	0.976
		WA	**16.52****	**21.2*****

Table S18: Mean richness (number of observed OTUs) and diversity (inverse Simpson Index) for plastic-associated and bulk soil bacterial communities in TN and WA, ± standard deviation.

Location	Treatment	Sample Type	Richness	Diversity Index
TN	BioAgri	Plastic	209 ± 14	10.028 ± 3.322
WA	BioAgri	Plastic	215 ± 32	13.443 ± 4.481
TN	Naturecycle	Plastic	210 ± 22	12.397 ± 3.833
WA	Naturecycle	Plastic	222 ± 20	14.055 ± 2.117
TN	Organix	Plastic	223 ± 17	11.555 ± 2.441
WA	Organix	Plastic	251 ± 28	21.138 ± 3.904
TN	PLA/PHA	Plastic	235 ± 17	9.641 ± 1.499
WA	PLA/PHA	Plastic	249 ± 6	14.625 ± 4.889
TN	Polyethylene	Plastic	265 ± 13	9.48 ± 5.066
WA	Polyethylene	Plastic	286 ± 14	22.348 ± 5.921
TN	Weedguard	Plastic	194 ± 8	10.041 ± 6.677
WA	Weedguard	Plastic	211 ± 15	19.354 ± 3.185
TN	BioAgri	Soil	314 ± 15	10.046 ± 0.849
WA	BioAgri	Soil	283 ± 4	10.01 ± 0.679
TN	Naturecycle	Soil	305 ± 9	10.466 ± 0.681
WA	Naturecycle	Soil	289 ± 5	9.246 ± 1.002
TN	Organix	Soil	303 ± 7	9.167 ± 0.477
WA	Organix	Soil	285 ± 11	9.293 ± 1.389
TN	PLA/PHA	Soil	298 ± 3	10.206 ± 1.287
WA	PLA/PHA	Soil	289 ± 8	10.001 ± 1.229
TN	Polyethylene	Soil	282 ± 15	8.875 ± 0.804
WA	Polyethylene	Soil	285 ± 11	10.348 ± 1.104
TN	Weedguard	Soil	302 ± 19	10.955 ± 1.639
WA	Weedguard	Soil	287 ± 5	11.154 ± 1.374

Table S19: Mean richness (number of observed OTUs) and diversity (inverse Simpson Index) for agriculturally-weathered plastic associated eukaryotic communities in TN and WA ± standard deviation.

Location	Treatment	Richness	Diversity Index
TN	BioAgri	55 ± 4	5.413 ± 1.596
WA	BioAgri	87 ± 6	12.97 ± 3.891
TN	Naturecycle	49 ± 3	2.951 ± 0.962
WA	Naturecycle	81 ± 5	12.29 ± 1.036
TN	Organix	55 ± 7	6.058 ± 1.401
WA	Organix	84 ± 10	9.886 ± 1.657
TN	PLA/PHA	52 ± 14	6.158 ± 3.467
WA	PLA/PHA	85 ± 6	12.9 ± 1.219
TN	Polyethylene	45 ± 8	8.173 ± 1.303
WA	Polyethylene	81 ± 8	11.19 ± 2.349
TN	Weedguard	44 ± 5	6.533 ± 1.562
WA	Weedguard	60 ± 10	5.516 ± 2.158

Table S20: F values from one-way ANOVAs testing differences in richness (number of observed OTUs) and diversity (inverse Simpson Index) between field mulch treatments and location for plastic-associated eukaryotic communities. Bold numbers and asterisks indicate significant differences between factor levels tested; ***p ≤ 0.001, ** p ≤ 0.01, *p ≤ 0.05.

Factor	Levels	Within	Richness F	Diversity Index F
Treatment	BioAgri, Naturecycle, Organix, PLA/PHA	TN	0.755	1.236
		WA	**6.132****	**6.31****
Location	TN, WA	Plastic	**45.16*****	**13.2****

Table S21: Percent contribution of individual taxa identified using SIMPER contributing up to 40% variation in microbial community composition between soil and agriculturally-weathered mulch associated bacterial communities in TN and WA.

Location	OTU number	Genus	% contribution
TN	28	*Methylobacterium*	14
	1	Bacteria_unclassified	11
	93	*Bacillus*	5
	20	*Sphingomonas*	6
	71	*Arthrobacter*	4
	38	*Deinococcus*	3
WA	38	*Deinococcus*	8
	1	Bacteria_unclassified	8
	29	*Hymenobacter*	7
	20	*Sphingomonas*	7
	28	*Methylobacterium*	5
	71	*Arthrobacter*	4
	35	*Comamonadaceae_unclassified*	4

References

Hayes, D.G., Wadsworth, L.C., Sintim, H.Y., Flury, M., English, M., Schaeffer, S., et al. (2017). Effect of diverse weathering conditions on the physicochemical properties of biodegradable plastic mulches. *Polymer Testing* 62**,** 454-467. doi: 10.1016/j.polymertesting.2017.07.027.

Sintim, H.Y., Bandopadhyay, S., English, M.E., Bary, A.I., DeBruyn, J.M., Schaeffer, S.M., et al. (2019). Impacts of biodegradable plastic mulches on soil health. *Agriculture, Ecosystems & Environment* 273**,** 36-49. doi: 10.1016/j.agee.2018.12.002.
